# Behavior of turnout sleepers in a large-scale ballast box test

**DOI:** 10.1038/s41598-025-01751-3

**Published:** 2025-05-14

**Authors:** Gernot Grohs, Paul Pircher, Martin Quirchmair, Harald Loy, Klaus Six, Ferdinand Pospischil

**Affiliations:** 1 Institute of Railway Infrastructure Design, Graz University of Technology, Graz, Austria; 2 Virtual Vehicle Research GmbH, Graz, Austria; 3 Getzner Werkstoffe GmbH, Bürs, Austria; 4 Department of Infrastructure, Unit of Intelligent Transport Systems, University of Innsbruck, Innsbruck, Austria

**Keywords:** Turnout sleeper, Pressure mapping, Track settlement, Sleeper-ballast interaction, Ballast box test, Cyclic loading, Long-term test, Railway, Civil engineering, Mechanical engineering

## Abstract

Turnouts are essential components of the railway infrastructure. Their sleepers change in length leading to asymmetric loading and structural discontinuities. This accelerates ballast wear and sleeper settlement creating challenges for maintaining track geometry and safety. Understanding load transfer from sleepers to ballast is key to improve railway durability and performance. This study examines the settlement behavior of turnout sleepers and the pressure distribution beneath them in a large-scale laboratory ballast box test. Cyclic loading tests were conducted on a long concrete sleeper with and without under sleeper pads (USP) to compare their load transfer characteristics and settlement behavior. For this laser displacement sensors and pressure mapping sensors (Getzner Sensor Sleeper technology) were used. A vertical cyclic load, oscillating between 5 kN and 160 kN at frequencies of 3 Hz to 5 Hz, was applied to one side of the turnout sleeper, while a constant load of 10 kN was applied to the opposite side to represent the structural stiffness of the turnout. For each test configuration more than four million load cycles were carried out. The sleeper showed a degressive settlement pattern with asymmetric distribution. The use of USP resulted in more uniform and gradual settlement, more uniform pressure distribution and reduction of pressure peaks over time. Additionally, the sleeper deformation caused by the load was mitigated and the occurrence of voids underneath the sleeper was significantly reduced. The sleeper with elasto-plastic USP show larger initial oscillation amplitudes due to the pad’s elasticity, which stabilizes over time.

## Introduction

Ballasted tracks have provided stable train operation for nearly 200 years. Most railways run on ballasted tracks. However, track geometry deteriorates over time as a result of varying settlement along the track. When changes in track geometry reach certain limits, maintenance is needed to restore track quality to its required level to ensure a safe train service.

A large part of the settlement can be attributed to the ballast layer^1^. Settlement cannot be easily modeled or predicted computationally or experimentally. Thus, the experimentally investigated settlement is often used as an estimation of the potential of settlement under certain conditions. There are many empirical models predicting ballast settlement depending on the number of train axle passes or cumulative load. These models tend to produce very different results. One reason could be a different consideration of the input parameters.

Maintenance of ballasted tracks is associated with high costs. Due to the increase in passenger and freight traffic, the usage of certain track sections has increased, leading to shortened maintenance cycles. Particularly areas with discontinuities, such as turnouts, crossings, and stiffness transitions, are prone to deteriorate faster.

A solid construction of the track design can help reduce maintenance requirements and increase service life. This leads to a reduction of service costs. To design better tracks, it is important to understand the behavior of ballast and sleeper interaction.

### Ballast layer

Turnout sleepers are critical components of the railway system, providing support for two tracks and transferring loads to the underlying ballast in the transition zone where two tracks merge into one. Unlike standard sleepers, turnout sleepers support two tracks and are subject to asymmetric loading during operation. Their dynamic response to repeated loading can result in differential settlement, posing a significant challenge to maintaining track quality and ensuring operational safety. The settlement of turnout sleepers in ballasted tracks is a complex phenomenon influenced by numerous factors, including the mechanical properties of the materials involved, the loading conditions from train traffic, and the interactions between the sleepers and the ballast.

One of the primary mechanisms contributing to the settlement of turnout sleepers is the deformation of the ballast layer beneath them. Simple changes of substructure design, such as increasing the degree of lateral confining pressure, shallower ballast shoulder slope, geogrids, or ballast modification can already help to improve the performance of the entire track^2–4^. The ballast, composed of coarse aggregates, is designed to provide stability and support for the sleepers. However, under the repeated passage of trains, the ballast can experience compaction, degradation, and movement, leading to uneven support conditions. Studies have shown that the quality and behavior of the ballast, as well as the sub-ballast and subgrade layers, play crucial roles in determining the extent of settlement experienced by the sleepers^5–7^. For instance, repeated loading can cause the ballast to deform permanently, resulting in some sleepers becoming partially or completely unsupported, which exacerbates the settlement issue^5,6,8^. Loading frequency is a crucial factor, as higher frequencies lead to greater settlement and other critical effects^9–13^.

The severity of track settlement is linked to the characteristics and performance of the ballast, sub-ballast, and subgrade^14^. The settlement process can be divided into two primary stages, the initial rapid phase that occurs after fresh ballast placement or maintenance tamping and a subsequent phase characterized by slow gradual ongoing subsidence. The gradual subsidence generally diminishes as traffic volume increases. The initial settlement phase can be attributed to the compaction of the ballast material. Once the ballast reaches a greater level of density, the subsequent phase of settlement gains prominence. This phase might be caused by particle breakage and particle rearrangement. It is determined by several factors, such as deviatoric stresses, subgrade stiffness, vibrations, and degradation. Settlement models often rely on logarithmic laws to describe settlement behavior, and the laboratory test results show that these models closely align with real-world observations^15^. However, the actual settlement is influenced by many factors that are not fully captured by these mathematical formulations. The stochastic nature of ballast settlement also significantly contributes to the variability in settlement behavior^16–18^. Several discrete element method (DEM) studies have been conducted to investigate the mechanical behavior of railway ballast^19–21^ and numerous experimental studies have also been performed^22–35^. Experimental setups, for example the ballast box, serve as valuable tools for validating simulation models.

### Sleeper

The mechanical properties of the sleepers themselves also affect their settlement behavior. Research indicates that the modulus of elasticity of the sleeper material and the sleeper support modulus play a significant role in determining how far the sleeper will settle into the ballast under load^36,37^. Uneven settlement can then lead to unsupported sleepers in the track. This phenomenon is particularly concerning in high-speed rail systems, where the dynamic loads are significantly higher, leading to increased rates of settlement and potential track failure^8,38^. The shape and length of the sleeper influence both settlement and the support condition^39^. During the initial phase of the vibration test, the difference in displacement amplitude between the sleeper ends and its center may indicate whether the support is predominantly central or located at the edges^40^.

Moreover, the design and maintenance practices employed in the construction of ballasted tracks can significantly influence the settlement behavior of turnout sleepers. Tamping operations, which involve compacting the ballast, are crucial for maintaining the integrity of the track structure. Studies have shown that effective tamping can enhance the mechanical properties of the ballast bed, thereby improving the support provided to the sleepers^41^. However, if tamping is not performed adequately, it can lead to the formation of voids beneath the sleepers, resulting in increased dynamic responses and further settlement^42^.

The interaction between the sleepers and the ballast is also critical in understanding settlement dynamics. When sleepers lose contact with the ballast, they can become “hanging,” which leads to increased vertical displacements and load redistributions among adjacent sleepers^43,44^. This situation can create a feedback loop, where the increased load on neighboring sleepers exacerbates their settlement, leading to a cascading effect throughout the track^44^. When a sleeper is hanging, it may not provide adequate support to the track and thus can lead to instabilities, uneven track alignment, and potential safety concerns for train operation. Some investigations were already conducted specifically on the distribution of loads beneath the sleeper^45–47^. Hanging sleepers can also enhance the dynamic behavior of the sleeper, leading to increased acceleration^48^. This acceleration has a significant impact on ballast settlement^49^. Consequently, monitoring and addressing the condition of the ballast and the support provided to the sleepers is essential for ensuring the long-term stability of railway tracks.

In summary, the settlement of turnout sleepers in ballasted tracks is influenced by a multitude of factors, including ballast degradation, sleeper material properties, dynamic loading conditions, and maintenance practices. The use of USPs and effective tamping operations can help mitigate some of the adverse effects associated with sleeper settlement. However, continuous monitoring and assessment of track conditions are necessary to prevent excessive settlement and ensure the safety and reliability of railway infrastructure.

### Under sleeper pads (USP)

Multiple studies have shown the benefits of under sleeper pads (USP) regarding maintenance reduction, track alignment, and life cycle costs in the past. Tamping intervals can be prolonged with USP, helping the operator to run the network in an economic way^50,51^.

USPs are designed to reduce the impact of dynamic loads, improve the load distribution across the ballast, and reduce ballast contact pressure^46,52–56^. By providing a cushioning effect, these pads can help maintain better contact between the sleepers and the ballast, thereby reducing settlement and the likelihood of differential settlement^1,56–59^. USPs are particularly effective in minimizing edge breakage of the ballast at the sleeper-ballast interface^1,60^.

USPs are made of different materials. Depending on the material and design, the USP displays elastic or elasto-plastic properties. Especially stiff elasto-plastic USP made of polyurethane are able to create a large contact area between ballast and sleeper and therefore reduce ballast contact pressure by more than 70%. USPs with similar stiffness but purely elastic properties are not able to achieve such reduction^61^.

The introduction of under sleeper pads (USP) has been proposed as a solution to mitigate the dynamic effects transmitted from the rails to the ballast for this test.

### Aim of this study

Several studies have investigated the behavior of ballast and the settlement of sleepers under cyclic loading, both numerically and experimentally. However, these studies have not specifically addressed turnout sleepers. Turnout sleepers differ significantly from standard sleepers in both geometry and loading conditions. Due to the design of the turnout, loads are primarily applied to one side of the sleeper, resulting in asymmetric load transfer to the ballast. This is in contrast to standard track sections, where sleepers typically experience more symmetric loading. The unique geometry of turnout sleepers, combined with asymmetric and often increased load conditions, justifies the need for dedicated investigation. These distinct loading behaviors can lead to different settlement patterns and support conditions that are not adequately represented in studies focusing solely on standard sleepers. The primary objective of this study is to gain a deeper understanding of the settlement behavior of turnout sleepers and the development of the pressure distribution beneath them. Pressure distribution is a crucial parameter for detecting the formation of hanging sleepers or cavities under sleepers. A key focus of this investigation is the influence of under sleeper pads (USP) on settlement, dynamic behavior, and pressure distribution. These experiments aim to enhance our understanding of the behavior and deterioration of turnouts and crossings, ultimately leading to improved measures for enhancing the performance and lifespan of railway tracks. The data collected will aid in predicting track alignment over time and provide insights into the formation and causes of cavities beneath sleepers which can be seen as hanging.

## Materials and methods

### Test setup

**Fig. 1:**
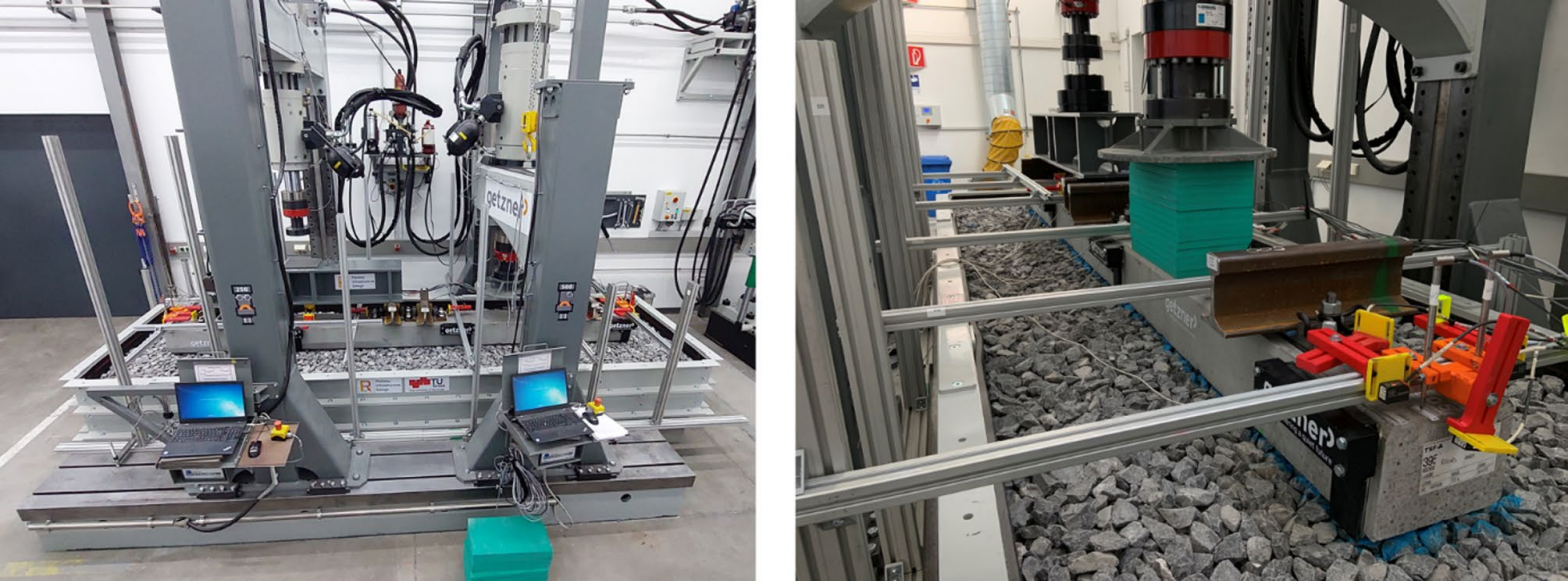
Test setup.

**Fig. 2:**
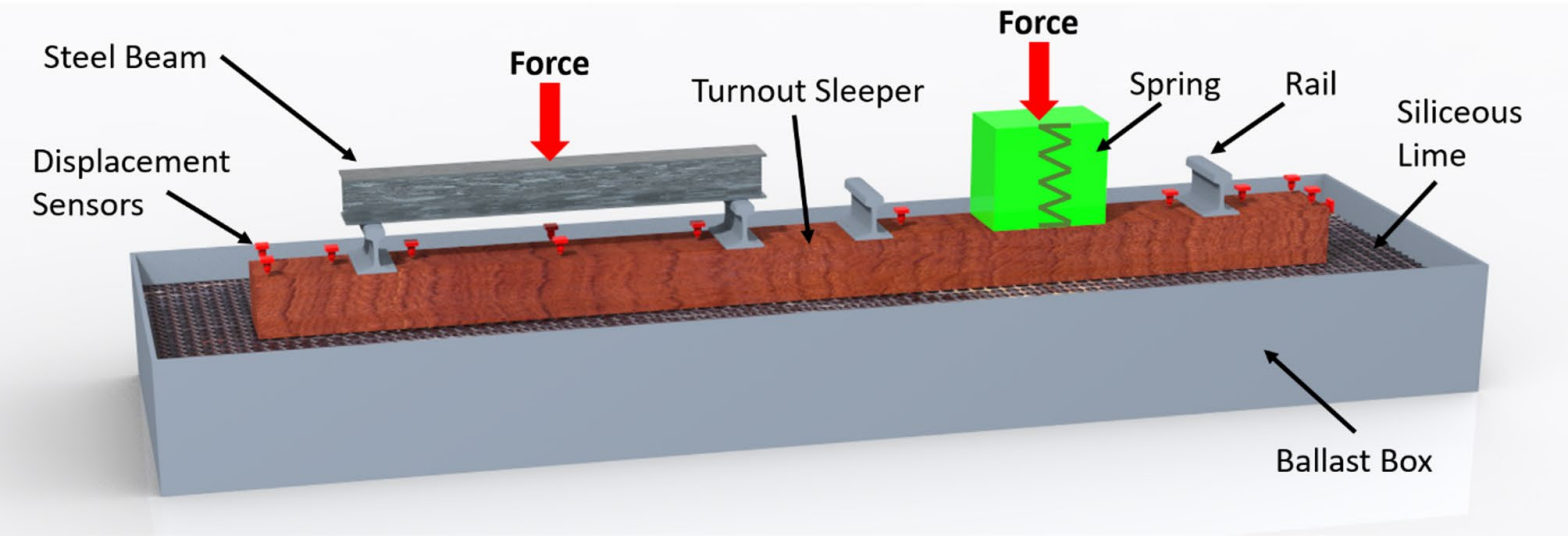
Sketch of the test configuration.

Figures 1 and 2 illustrate the setup of the ballast box test. In this configuration, the force is applied to the left rail track and distributed evenly across both rails. The hydraulic cylinder generating the force is connected to the sleeper’s load application point via a spherical seating, which allows a certain amount of tilting. To simulate the behavior of the adjacent track and achieve more realistic results, the right track is subjected to a constant preload of 10 kN. This force is applied using a hydraulic cylinder, with an elastic element positioned between the cylinder and the sleeper to provide a defined preload and holding of the sleeper.

The laboratory test represents a simplified approximation of real-world conditions and is intended to provide initial insights into the behavior of turnout sleepers under asymmetric loading. While this setup cannot fully capture the complexity of field conditions, it allows for a controlled investigation of fundamental load transfer mechanisms and settlement behavior, forming a basis for future, more comprehensive studies.

### Ballast box

**Fig. 3:**
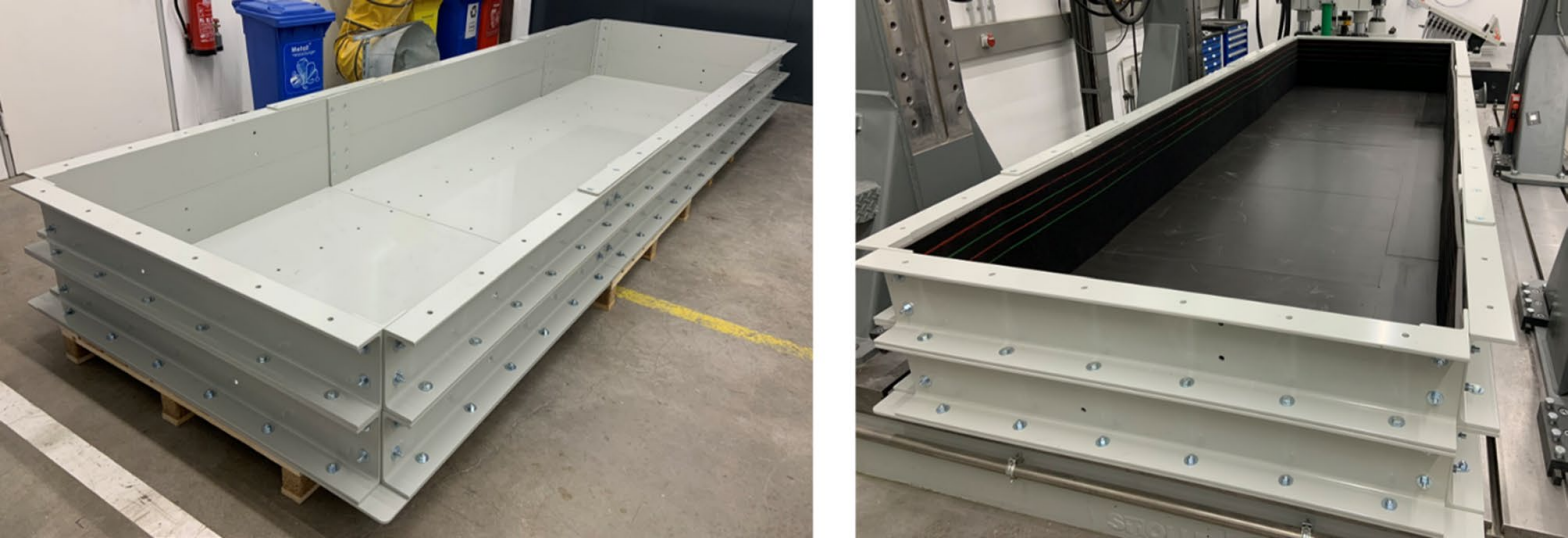
Steel ballast box and damping material.

A steel box, as shown in Fig. 3, with dimensions of 5 m x 1.5 m x 0.5 m, was used to contain the ballast and sleeper for the settlement test. The interior side surfaces of the box were lined with damping material to mitigate dynamic wave reflections at the edges, effectively reducing noise and suppressing oscillations within the system. Ecomer ER 607, with a thickness of 17 mm and a static bedding modulus of 0.12 N/mm^3^ was used for this purpose.

Additionally, the box dimensions were designed to be sufficiently large to minimize wave deflection. An elasto-plastic under sleeper pad (USP) was installed at the bottom of the box to simulate the behavior of the subsoil. Sylomer SLB 3007G, with a thickness of 12 mm and a static bedding modulus of 0.36 N/mm^3^ was used for this purpose.

### Ballast

**Fig. 4:**
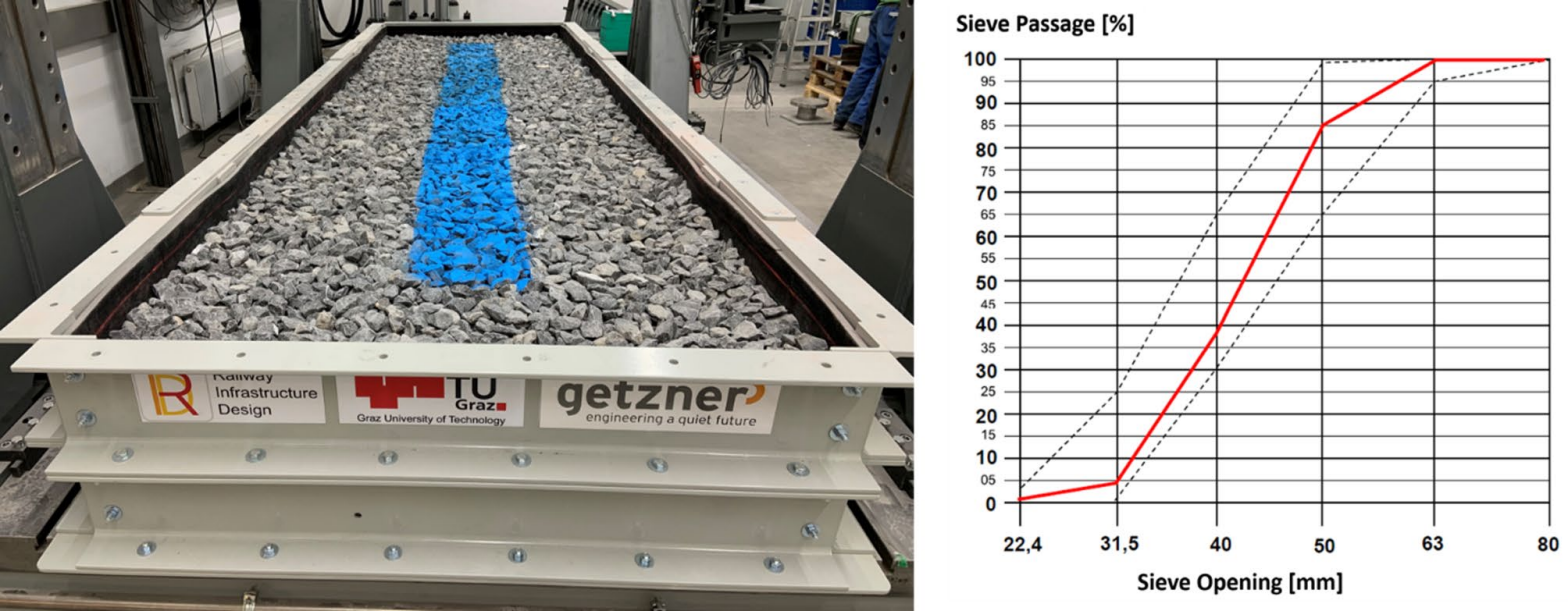
Steel box filled with ballast (left) and ballast sieve curve (right).

Siliceous limestone was used as the ballast material. The solid (particle) density of the ballast was approximately 2680 kg/m^3^, while the bulk density of the compacted ballast layer was around 1950 kg/m^3^. As shown in Fig. 4, the ballast was sieved prior to insertion. The ballast was applied in three layers, each 10–15 cm thick, with each layer compacted using a vibrating soil compactor plate (Dynapac LF62) to achieve a uniform and stable ballast bed. Prior to the main experiment, preliminary tests were conducted to assess and adjust the compaction procedure, ensuring that the ballast bed achieved a level of compactness representative of field conditions. While exact field compaction levels can vary depending on location and traffic history, care was taken to replicate realistic stiffness and support characteristics as closely as possible within the laboratory setting. For both experiments, fresh ballast was used, and the box was refilled and compacted accordingly. The total height of the ballast layer was 35 cm. Blue markings were applied to indicate the sleeper position. These markings also facilitated the observation of ballast stone breakage or realignment of ballast stones after the test (Fig. 4).

### Sleeper and USP

**Fig. 5:**
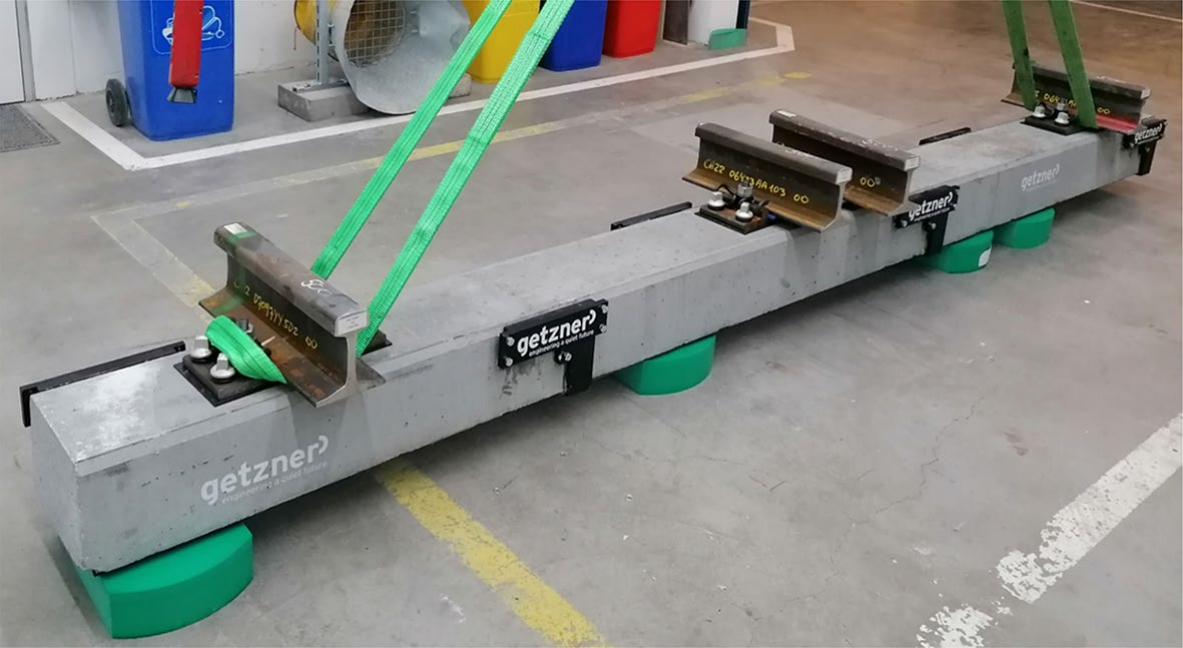
TSF-A turnout sleeper.

A TSF-A turnout sleeper (Fig. 5) was used in the experiment, with dimensions of 4.206 m × 0.3 m × 0.22 m and a weight of 727 kg. This sleeper is designed to support two tracks side by side. The experiment was conducted twice: once with the sleeper equipped with under sleeper pads (USP) and once without them. For the USP material Sylomer SLB 3007G, with a thickness of 12 mm and a static bedding modulus of 0.36 N/mm^3^ was used. The applied load was transferred from the hydraulic cylinder to the rails via a steel beam. The steel beam weighs about 300 kg and each of the four rail pieces weighs 30 kg. In total there is 1147 kg, resulting in 11.25 kN additional force acting beneath the sleeper. The weight of the steel beam was not included in the applied load and therefore acted as an additional static force on the system.

### Load generator

A hydraulic load generator, as shown in Fig. 1, was used in the experiment. The system consisted of a rigid frame and two hydraulic cylinders designed to apply loads to the sleeper. To accommodate tilting caused by inhomogeneous settlement along the sleeper, a spherical seating was incorporated at the cylinder, allowing slight tilting at the load transfer point.

### Sensors

**Fig. 6:**
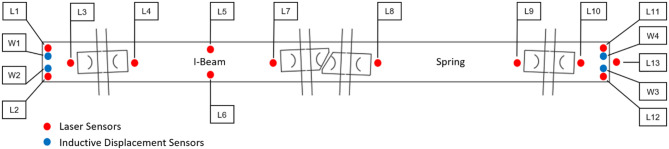
Displacement sensor plan of the sleeper (top view).

**Fig. 7:**
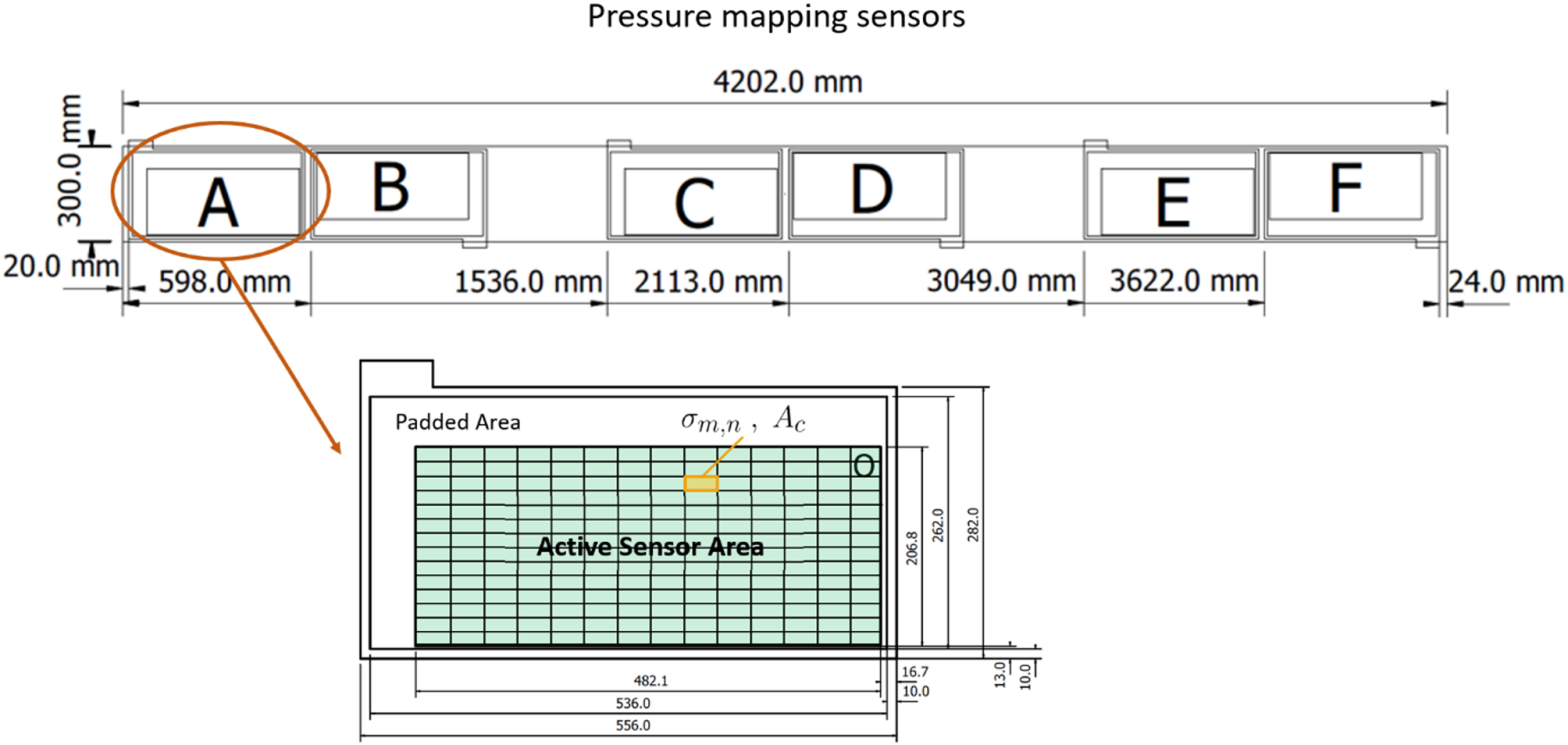
Pressure mapping sensor plan of the sleeper (top view)^62^.

**Fig. 8:**
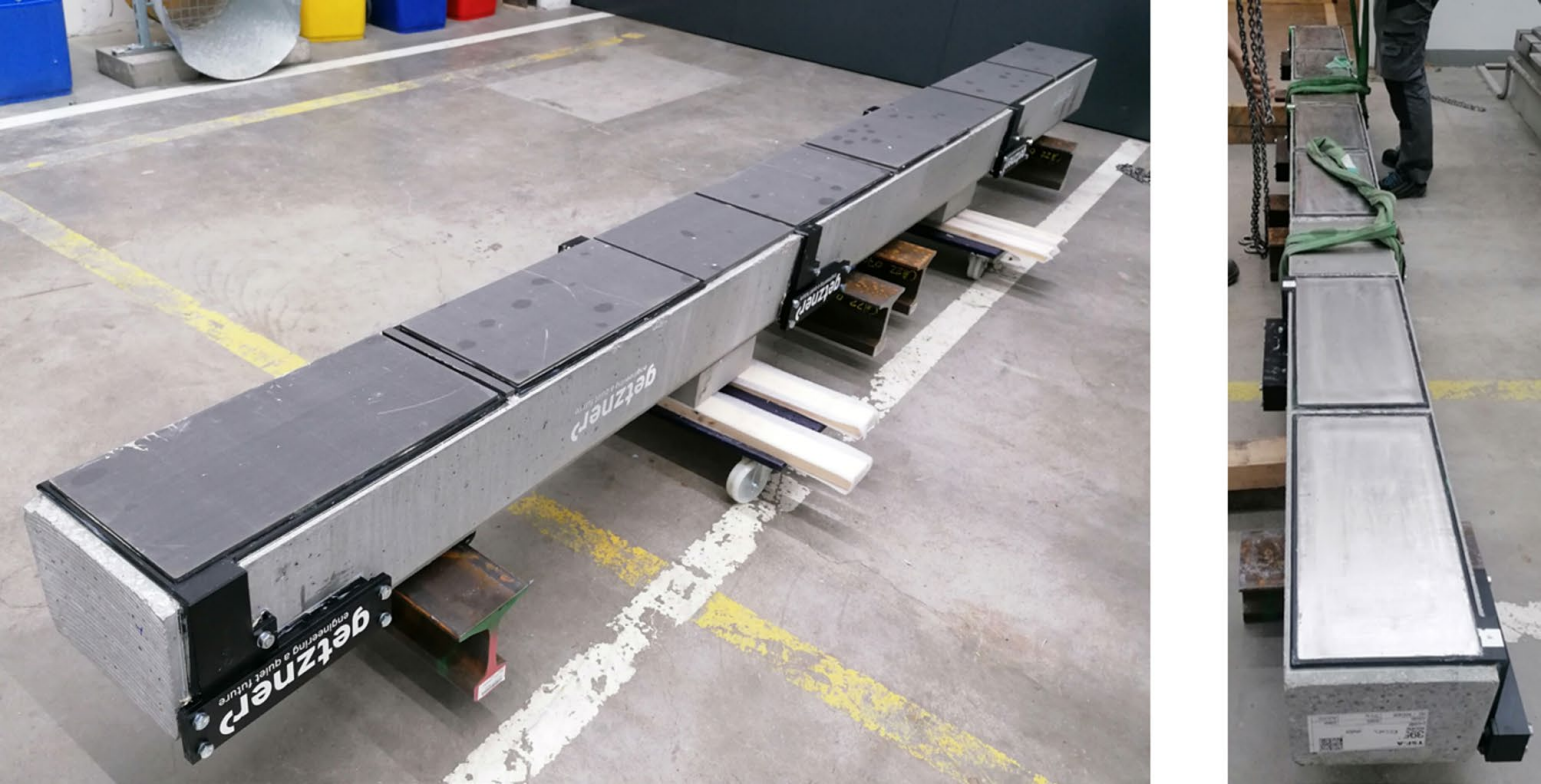
Bottom of sleeper with and without USP.

The sleeper was equipped with several laser sensors to record the change of position and orientation due to cyclic loads. Additionally, inductive displacement sensors were installed on the sides of the sleeper to verify the measured values of the laser sensors. Figures 6 and 7 show the sensor configuration layout. The sensors attached in the middle of the sleeper are for the detection of sleeper deformation due to the applied force. This provides important insight into the development of “hanging sleepers.” Furthermore, the load distribution underneath the sleeper was detected with a Getzner Sensor Sleeper specifically developed for this field of use to obtain better knowledge of ballast contact pressures. The sensor pads are not capable of covering the entire underside of the sleeper because of the design and the electronic parts located on the sides. Figure 8 shows the arrangement of the Getzner Sensors with and without USP. The sensitivity of the sensors is chosen to fully cover the peak pressures at the sleeper-ballast interface, which indirectly leads to imprecise measuring of lower pressure values. Hence, the measured pressure values only allow for a qualitative analysis.

For pressure mapping, all measurements were conducted with consistent sensor sensitivity for each sleeper. The sensitivity was adjusted for each sleeper to ensure alignment with the appropriate measuring range. The sensitivity of the sensor pads determines the range within which the sensors can provide accurate measurements. Given that the sensor has a resolution of 8 bits, it is crucial to define the measurement range appropriately to ensure accurate data collection. Values falling below or exceeding this range are truncated, limiting the sensor’s ability to measure accurately at extreme pressures. Consequently, the measured forces are approximations rather than precise values. The plots were generated using the same scale to facilitate easy comparison.

It is important to note that the active sensor surface covers approximately half (47.45%) of the total surface area of the turnout sleeper. As a result, while the total force distribution across the entire sleeper surface cannot be fully determined, the sensor data still provides valuable insights into the pressure distribution patterns.

### Test procedure

The ballast was inserted in the box layer by layer. Each layer was compacted individually with a low-destructive vibrating plate. Afterwards the sleeper was placed on the ballast with a crane. The sleeper was not buried in the ballast like conventional operating rail track sleepers. The load distribution on the two rails was equally distributed to the rails via a steel beam lying on the two left rails constituting the turnout’s through route.

Preliminary tests were conducted using static and cyclic loads up to a maximum of 120 kN, with both asymmetric and symmetric load distributions applied to the rail. In these tests, one side of the sleeper was either preloaded or loosened, depending on the test scenario. All tests were performed at a maximum load frequency of 3 Hz. However, these preliminary experiments are not the primary focus of this paper and will not be discussed in detail. The focus of this study is on long-term cyclic testing, which is challenging to study numerically. It is important to highlight that before the long-term cyclic tests were conducted, the experimental setup with under sleeper pads (USP) was preconditioned with approximately 200,000 cycles, while the setup without under sleeper pads (no USP) underwent preconditioning with about 140,000 cycles.

At the beginning of the experiment, the sleeper requires a certain period to stabilize, which can be characterized as a “run-in” condition. The pre-tests can be considered as the run-in phase. Following this stabilization phase, the long-term cyclic loading test can commence.

The core of this study is the long-term cyclic loading test, in which the sleeper was subjected to an oscillating force ranging from 5 kN to 160 kN, with frequencies varying between 3 Hz and 5 Hz. In the test involving under sleeper pads (USP), the frequency was increased from 3 Hz to 5 Hz after approximately 1.3 million cycles. Conversely, in the test without USP, the frequency adjustment from 3 Hz to 5 Hz occurred after approximately 1.8 million cycles.

## Results

### Sleeper settlement

**Fig. 9:**
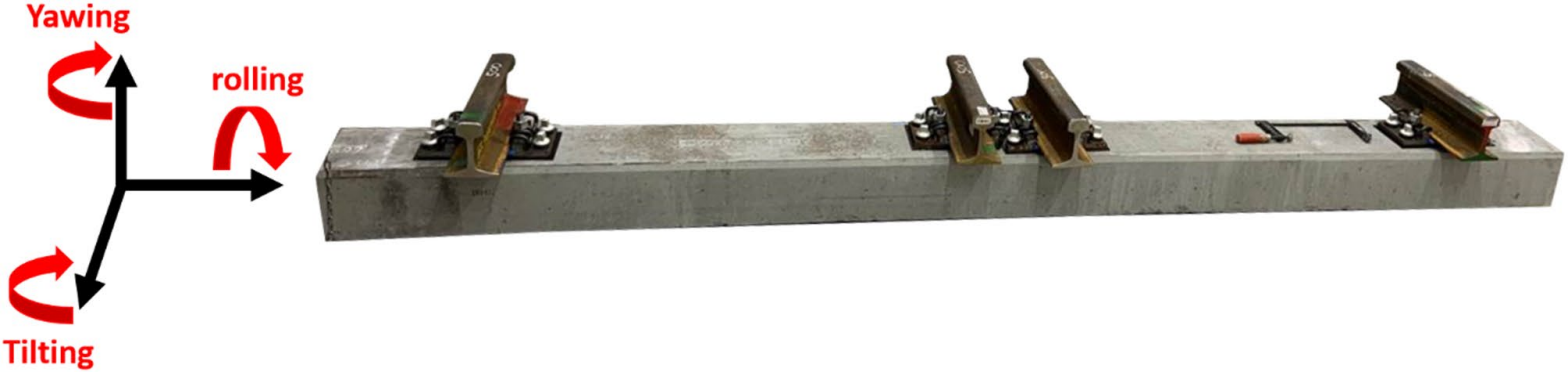
Definition of sleeper movement.

**Fig. 10:**
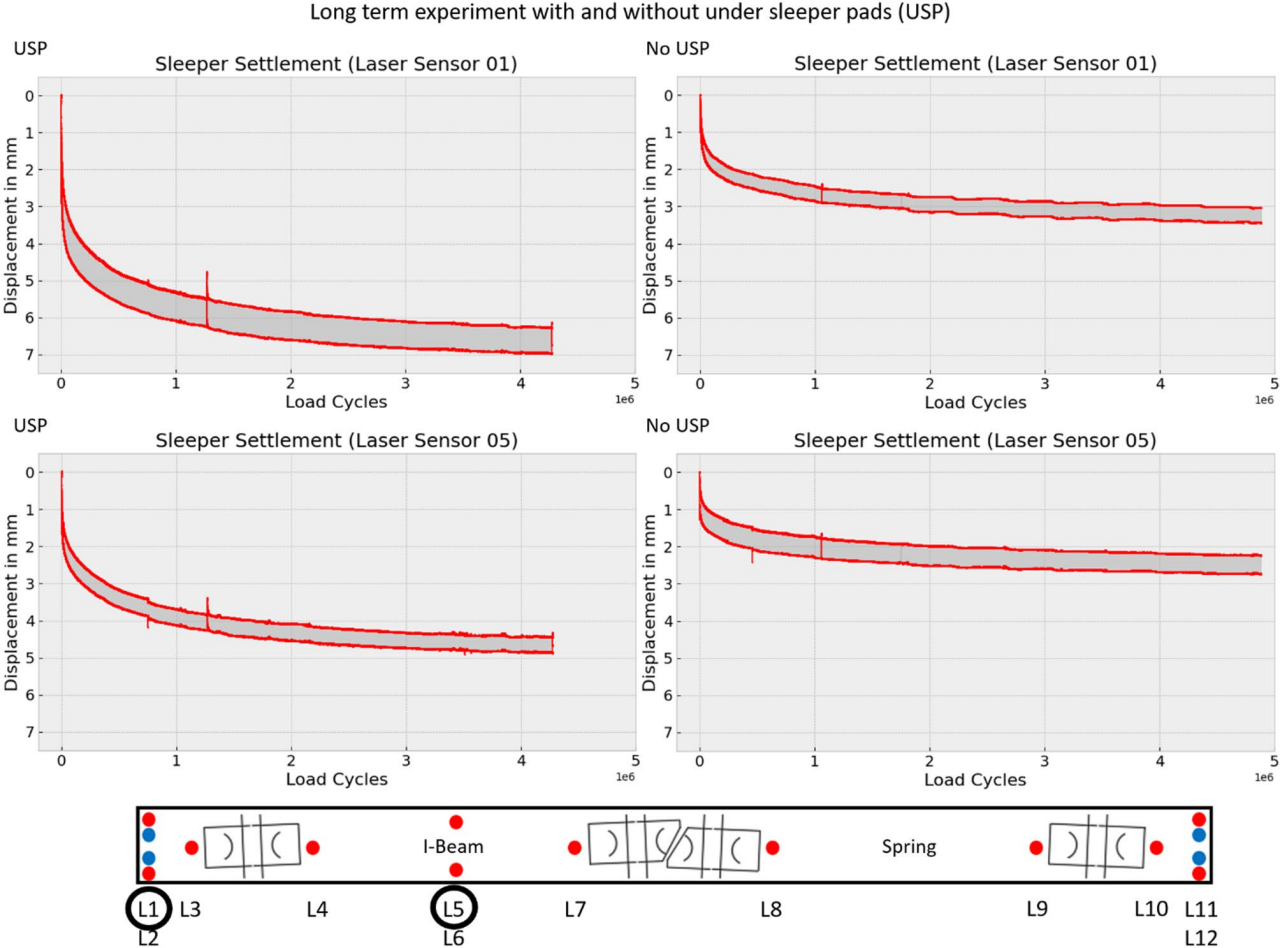
Sleeper settlement for laser sensor 1 and 5 with and without USP.

Figure 9 presents the movement coordinates of the sleeper. For the analysis, two critical points on the sleeper were selected: One is located on the far left, which is considered as the point with the greatest movement and settlement, and another one is located directly beneath the loaded track on the left side (Fig. 10). Figure 10 illustrates the sleeper’s settlement behavior as represented by the data of sensor 1 and sensor 5. A degressive settlement pattern is clearly observed, with the majority of settlement occurring during the initial phase of the experiment. Over time, the settlement rate appears to converge toward a final value. Preliminary tests suggest that this degressive settlement pattern is repeated when the applied force is increased. The diagram represents an enveloping curve that defines the upper and lower bounds of force variation during oscillation.

**Fig. 11:**
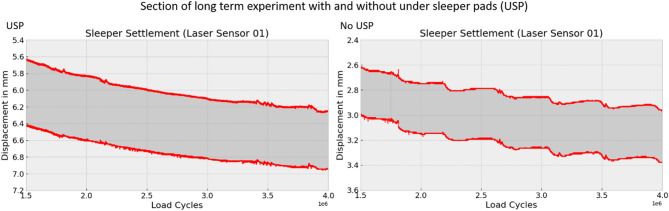
Detail view of settlement for laser sensor 1 for USP and no USP.

The settlement curve for sleepers without USP is characterized by increased irregularities and numerous discontinuities, as shown in Fig. 11. This behavior can be attributed to the more direct contact between the sleeper and ballast in the absence of USP, resulting in significantly higher surface pressures at the contact points and higher breakage or rearrangement of the ballast. In contrast, the presence of USP promotes a more homogeneous pressure distribution and milder pressure gradients, preventing individual stones from embedding deeply into the ballast. Additionally, the ballast stones can penetrate the elasto-plastic under sleeper pad (USP) material, leading to interlocking.

**Fig. 12:**
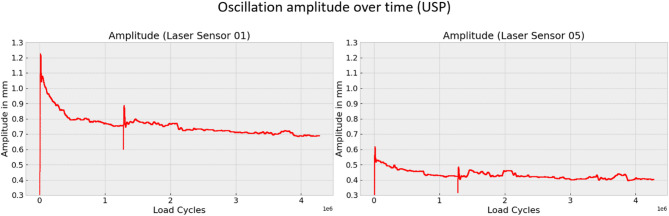
Oscillation amplitude at laser sensor 1 and 5 for sleeper with USP.

**Fig. 13:**
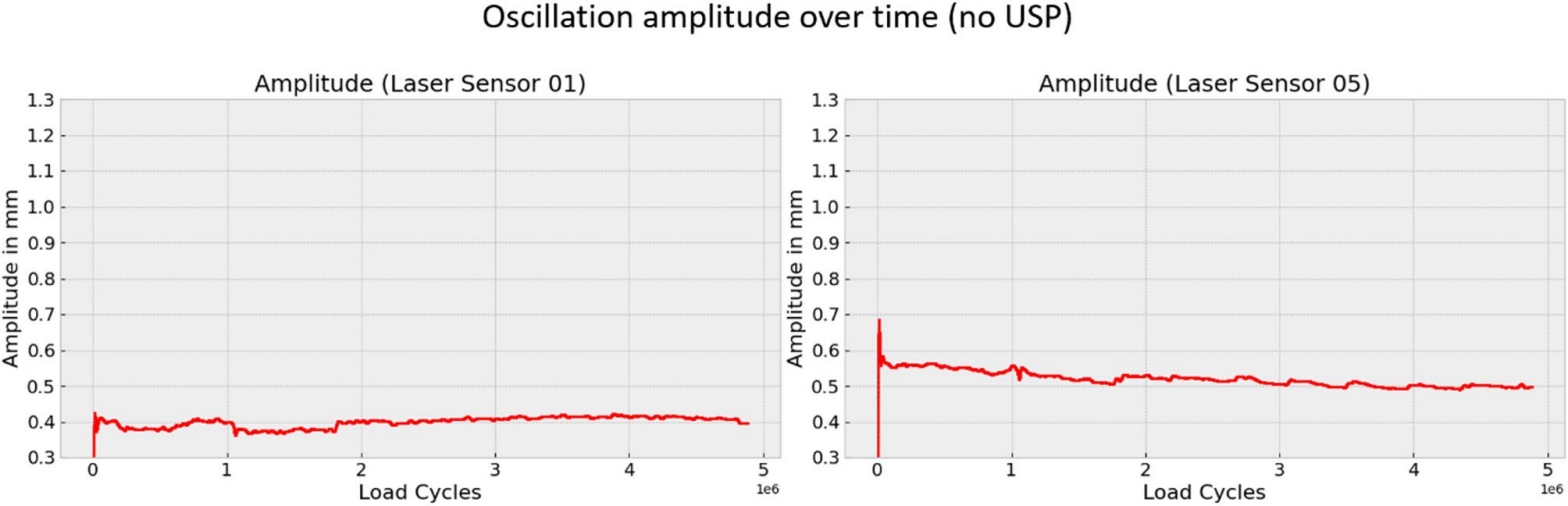
Oscillation amplitude at laser sensor 1 and 5 for sleeper without USP.

The under sleeper pads (USP) resulted in both higher total displacement and greater oscillation amplitude, as seen in Figs. 10, 12 and 13. On the far left side, the total displacement of the sleeper with USP is approximately twice as large as that of the sleeper without USP, with a similar doubling in oscillation amplitude. Directly beneath the loaded track, the total displacement of the sleeper with USP is also about twice as high as for the sleeper without USP. However, in this location, the oscillation amplitude of the sleeper without USP is significantly higher than that of the sleeper with USP.

The under sleeper pad (USP) exhibits both elastic and plastic behavior, depending on the magnitude and duration of the applied force. Preliminary tests indicated that the pad material requires additional time to recover and expand. Consequently, the settlement and alignment of the sleeper with USP may change slightly over time when subjected to lower forces or no load at all. The laser sensors measure the position and movement of the sleeper; however, they are unable to differentiate between deformation of the ballast and deformation of the under sleeper pad (USP).

Changes in oscillation amplitude, in particular, can reveal important information about the dynamic behavior of the sleeper. Figures 12 and 13 show the oscillation amplitude over time at two positions on the sleeper, comparing the sleeper with and without USP. The data reveal that, for sleepers with USP, the oscillation amplitude on the far left side of the sleeper decreases over time, whereas for the sleeper without USP, the amplitude remains relatively constant. This phenomenon may be attributed to the elasto-plastic pad material, which gradually compresses over time.

### Sleeper position and contact points

The alignment changes of the sleeper were analyzed by evaluating displacement sensor data at specific time points or cycle numbers. These sensors, positioned at various locations along the sleeper, also provided insight into the sleeper’s deformation under load.

**Fig. 14:**
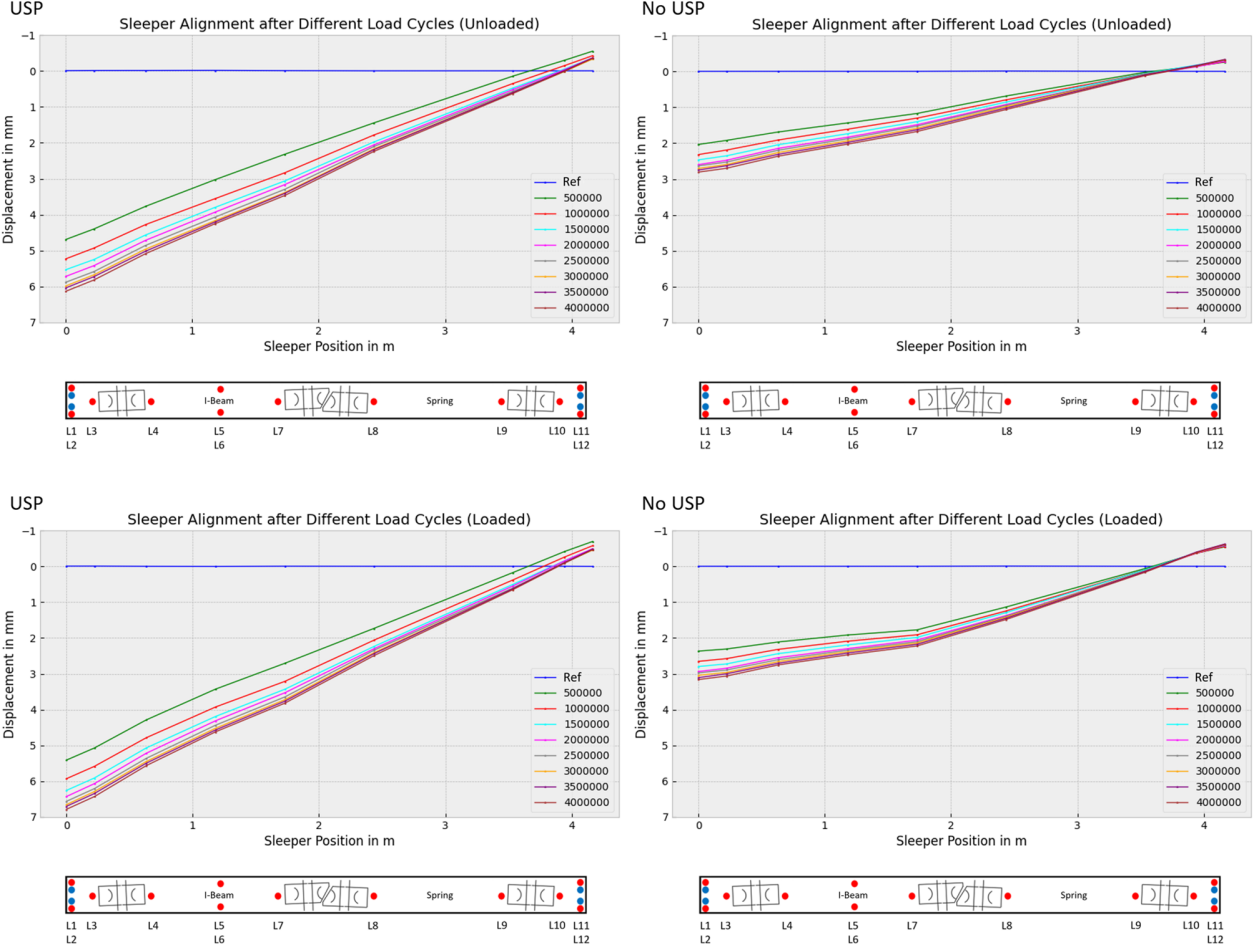
Alignment of turnout sleeper under varying load conditions after different load cycles.

Figure 14 shows the alignment of the sleeper after a different number of load cycles, where on the left side the sleeper without USP and on the right side the sleeper with USP is shown. For positions where two sensors were located at the same lateral position, the mean value of their measurements was used since the investigation of sleeper rolling was not the aim of this study. In the following, the “unloaded” condition corresponds to a force of 5 kN, while the “loaded” condition corresponds to a force of 160 kN. The lower force is meant to represent the preload typically observed under field conditions. In Fig. 14 the top rows show the “unloaded” and the bottom row the “loaded” condition.

**Fig. 15:**
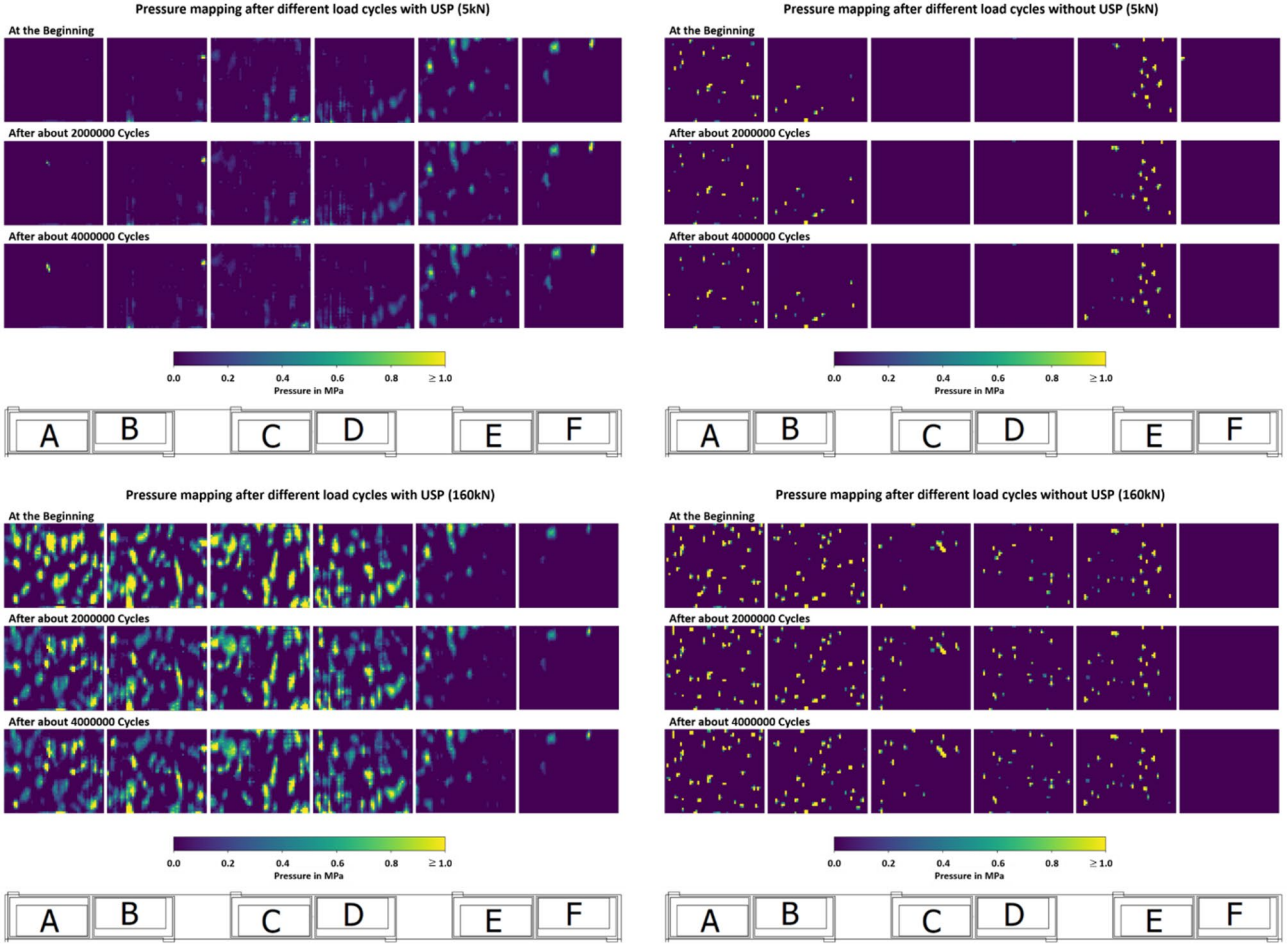
Pressure mapping for sleeper with and without USP after different number of load cycles.

The data shows a degressive settlement pattern. What can also be seen is that the sleeper gets tilted so strongly that the other end of the sleeper moves in the opposite direction. That means that this side gets relieved while the other side gets more load. Further, the sleeper without USP exhibits slight bending under the loaded condition, a phenomenon not observed in the sleeper with USP. This difference can be attributed to the softer and more homogeneous embedding provided by the USP. In contrast, the sleeper without USP does not exhibit smooth embedding. Instead, it rests on discrete bearing points that act as abutments. Between these points, the sleeper experiences bending under load. The pressure distribution indicates the presence of a “hanging sleeper” effect (Fig. 15). Given that concrete is inherently weak in resisting tensile and bending forces, this condition can result in accelerated fatigue, cracking, and wear of the sleeper. To gain deeper insights, a more detailed examination of the data is conducted in the following sections.

Figure 15 illustrates the pressure distribution on the sleeper at different load cycles in the loaded and unloaded state. Three stages are depicted: the beginning, middle (about 2 Mio cycles), and end of the long-term test (about 4 Mio cycles). In the unloaded state of the sleeper with USP, the pressure distribution is skewed toward the right side, likely due to the initial preload of 10 kN applied on that side. When the sleeper is loaded, the pressure distribution shifts more toward the left side. The distribution becomes more pronounced under load, with the individual ballast stones pressing through the USP onto the sleeper becoming clearly visible. For the sleeper without USP, the pressure gradients are significantly higher, and the contact areas are considerably smaller. As a result, the peak pressures are considerably higher compared with the setup with USP. Over time, the pressure distribution for the sleeper without USP indicates the development of new contact points. For the sleeper with USP, the high pressure contact points decrease over time, suggesting a redistribution of forces along the sleeper. The contact zones beneath the sleeper with USP remained largely unchanged, indicating that no significant voids or unsupported areas developed over time.

It can be clearly seen that for the sleeper without USP there is an unsupported area in the middle of the sleeper. Only after the force is applied does the sleeper’s middle part touch the ballast. This leads to unfavorable loading conditions for the sleeper which can entail high bending stress in the long run.

### Pressure distribution under the sleeper

The pressure distribution beneath the sleeper provides valuable insights into the sleeper’s embedding and contact conditions, which are key factors influencing settlement behavior. The load distribution underneath the sleeper is not homogeneous due to the ballast. High contact forces caused by individual ballast stones carrying the sleeper can lead to higher wear and breakage of the ballast stones and therefore change the bedding conditions of the sleeper. Uneven pressure distribution can lead to unsupported or hanging sleepers and increased stress on the sleeper. To better understand the physics of the sleeper-ballast interaction, a detailed analysis of the pressure distribution is conducted. This analysis focuses on maximum pressure values, mean pressures, distribution patterns, and the standard deviation of the measured pressures.

Most of the force is transmitted through individual stones beneath the sleeper. The concentration of high forces on these individual stones may cause them to penetrate deeper into the ballast layer, resulting in reorientation, rearrangement, or even breakage. This process could lead to sudden changes in the sleeper’s position and alignment, as shown in Fig. 11. Fewer contact points with concentrated high forces appear to exacerbate this phenomenon. The USP primarily helps distribute the applied forces across a larger area of the sleeper surface and over a greater number of individual ballast stones, thereby reducing the maximum pressure.

**Fig. 16:**
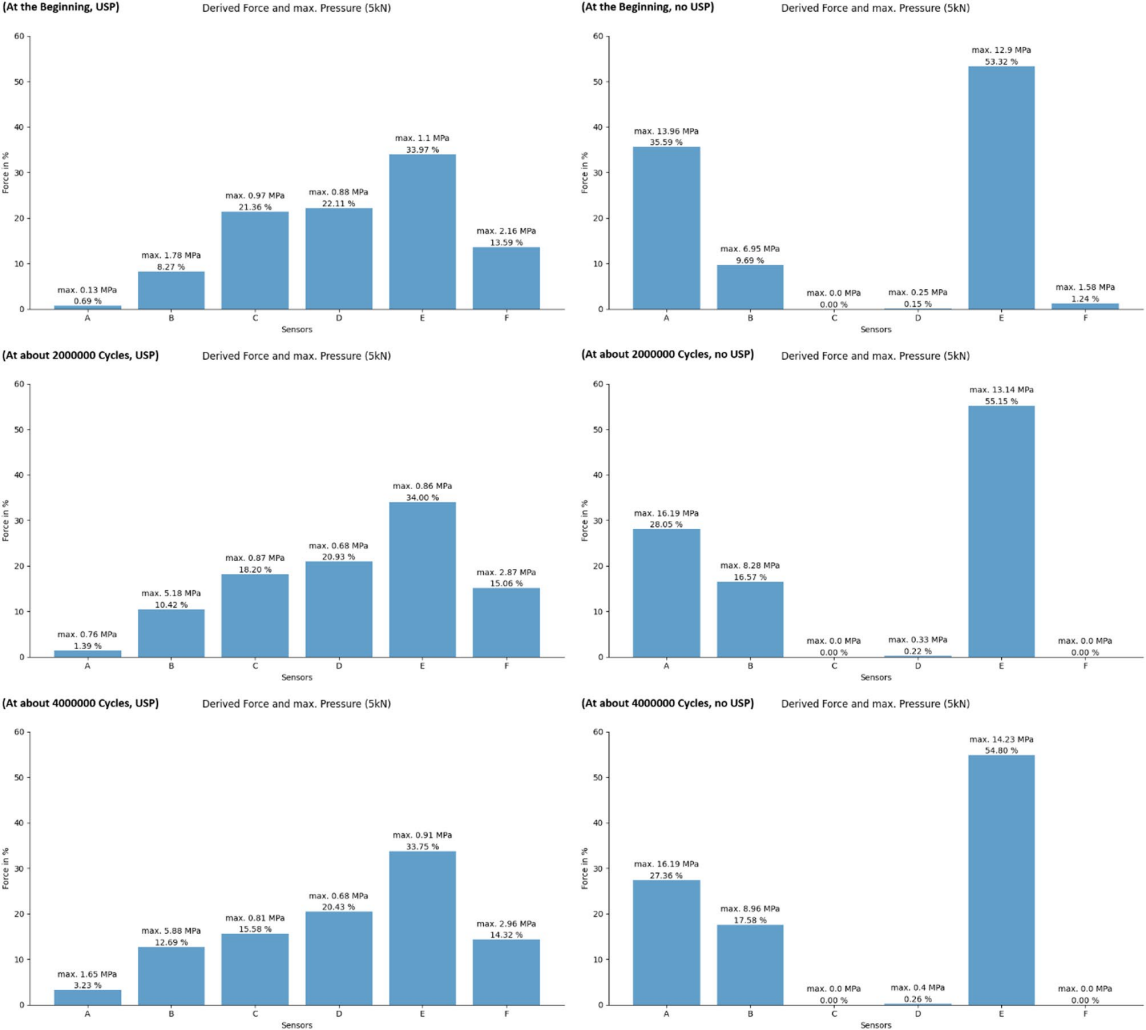
Force distribution underneath the sleeper at low force state.

**Fig. 17:**
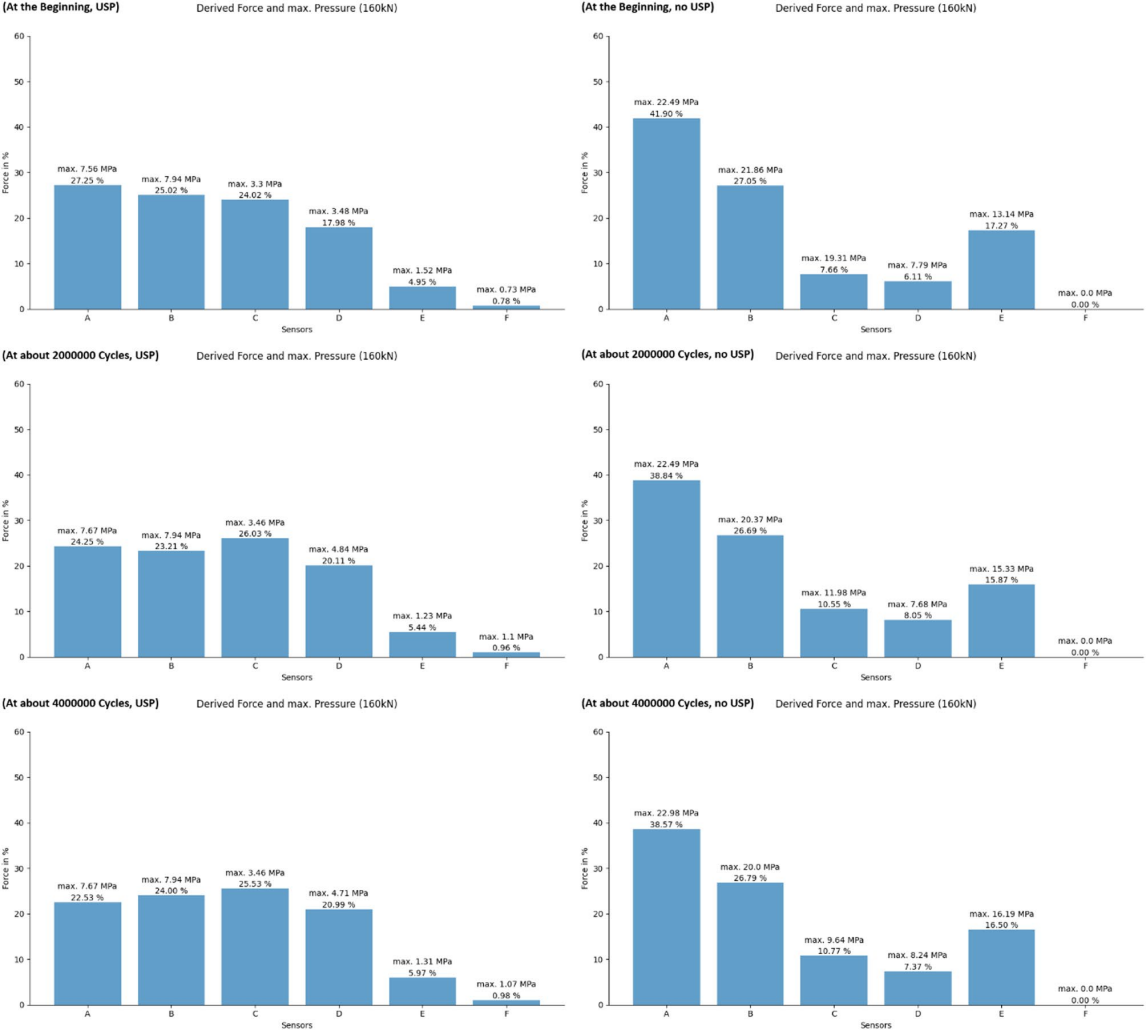
Force distribution underneath the sleeper at high force state.

Figures 16 and 17 illustrate the estimated derived forces through different sleeper zones and the maximum pressure recorded by each sensor at the three different stages of the experiment. Accurately measuring the total accumulated force requires sensors capable of precisely detecting pressures across a wide range. The figures presented represent an estimated percentage of the force detected by each sensor rather than the absolute total forces. These diagrams correspond well with the sleeper alignments shown earlier (Fig. 14) and help explain the bending observed in the sleeper without USP, which is caused by the unsupported area in the middle. Also, a slight redistribution of contact forces is observed. Regions subjected to higher initial forces tend to exhibit a decrease in force over time, whereas areas with lower initial forces often experience an increase. This balancing process aims to achieve a more uniform distribution of forces across the sleeper surface, promoting more even load transfer.

There is a clear difference in the force distribution between sleepers with and without USP. Sleepers equipped with a USP show a more uniform and predictable force distribution, with reduced sensitivity to the flatness and arrangement of the ballast layer beneath them. In contrast, sleepers without a USP are more strongly influenced by the variability and unevenness of the ballast layer. The USP has the ability to equalize these contact points due to its deformable elastic behavior, unlike the rigid concrete of the sleeper.

Tables 1 and 2 provide statistical insights into the pressure distribution beneath the sleeper. The data reveal for both sleepers that the majority of pressures are concentrated near zero, with a small standard deviation. Only a few pressure points are significantly higher than the rest.

The sleeper equipped with under-sleeper pads (USP) exhibits a reduction in the mean pressure values in the loaded state, particularly in the higher-loaded areas (A, B, C). In contrast, this reduction is not as evident for the sleeper without USP.

In the loaded state, the standard deviation of pressure tends to decrease over time for both sleepers with and without under-sleeper pads (USP). This suggests that the pressure becomes more evenly distributed across the sleeper surface as the number of cycles increases.

**Table 1:** Statistical data of pressure mapping for sleeper with USP.

	A		B		C		D		E		F	
	5 kN	160 kN	5 kN	160 kN	5 kN	160 kN	5 kN	160 kN	5 kN	160 kN	5 kN	160 kN
Maximum pressure (USP)												
Start	0.13	7.56	1.78	7.94	0.97	3.3	0.88	3.48	1.1	1.52	2.16	0.73
≈ 2 Mio	0.76	7.67	5.18	7.94	0.87	3.46	0.68	4.84	0.86	1.23	2.87	1.1
≈ 4 Mio	1.65	7.67	5.88	7.94	0.81	3.46	0.68	4.71	0.91	1.31	2.96	1.07
Mean pressure (USP)												
Start	0.00068	0.251	0.00815	0.230	0.02107	0.221	0.02181	0.165	0.03350	0.045	0.01340	0.007
≈ 2 Mio	0.00131	0.186	0.00988	0.178	0.01727	0.200	0.01985	0.154	0.03226	0.041	0.01429	0.007
≈ 4 Mio	0.00331	0.167	0.01304	0.178	0.01601	0.190	0.02100	0.156	0.03468	0.044	0.01471	0.007
Standard deviation of pressure (USP)												
Start	0.00810	0.482	0.06148	0.565	0.07019	0.388	0.06960	0.307	0.11368	0.153	0.09704	0.053
≈ 2 Mio	0.01963	0.465	0.12541	0.527	0.05812	0.331	0.06027	0.277	0.09763	0.127	0.11206	0.055
≈ 4 Mio	0.05181	0.442	0.14423	0.550	0.05542	0.317	0.06262	0.277	0.10344	0.133	0.11495	0.055

**Table 2:** Statistical data of pressure mapping for sleeper without USP.

	A		B		C		D		E		F	
	5 kN	160 kN	5 kN	160 kN	5 kN	160 kN	5 kN	160 kN	5 kN	160 kN	5 kN	160 kN
Maximum pressure (no USP)												
Start	13.96	22.49	6.95	21.86	0.0	19.31	0.25	7.79	12.9	13.14	1.58	0.0
≈ 2 Mio	16.19	22.49	8.28	20.37	0.0	11.98	0.33	7.68	13.14	15.33	0.0	0.0
≈ 4 Mio	16.19	22.98	8.96	20.0	0.0	9.64	0.4	8.24	14.23	16.19	0.0	0.0
Mean pressure (no USP)												
Start	0.05032	0.214	0.01369	0.138	0.0	0.039	0.00021	0.031	0.07539	0.088	0.00175	0.0
≈ 2 Mio	0.03613	0.191	0.02134	0.131	0.0	0.052	0.00028	0.039	0.07104	0.078	0.0	0.0
≈ 4 Mio	0.03686	0.187	0.02368	0.129	0.0	0.052	0.00034	0.035	0.07383	0.080	0.0	0.0
Standard deviation of pressure (no USP)												
Start	0.54057	1.373	0.19842	0.991	0.0	0.519	0.00738	0.320	0.74221	0.777	0.04737	0.0
≈ 2 Mio	0.47357	1.283	0.28657	0.957	0.0	0.491	0.00975	0.373	0.69622	0.751	0.0	0.0
≈ 4 Mio	0.48109	1.261	0.31130	0.937	0.0	0.481	0.01182	0.351	0.72103	0.771	0.0	0.0

### Redistribution of supporting forces

The statistical analysis of the pressure mapping data gives more precise insights into the pressure distribution beneath the sleeper. The data reveals for both sleepers that the majority of pressures are concentrated near zero, with a small standard deviation. Only a few pressure points are significantly higher than the rest.

The maximum pressure values can fluctuate in both directions, either increasing or decreasing. A potential explanation for the decrease in pressure is the rearrangement or breakage of ballast particles, which redistributes the load. On the other hand, an increase in pressure can occur due to uneven settlement, where certain ballast stones penetrate deeper into the sleeper, concentrating the load in specific areas.

The sleeper equipped with under sleeper pads (USP) exhibits a reduction in the mean pressure values in the loaded state, particularly in the higher-loaded areas (A, B, C). In contrast, this reduction is not as evident for the sleeper without USP.

In the loaded state, the standard deviation of pressure tends to decrease over time for both sleepers with and without under sleeper pads (USP). This suggests that the pressure becomes more evenly distributed across the sleeper surface as the number of cycles increases.

**Fig. 18:**
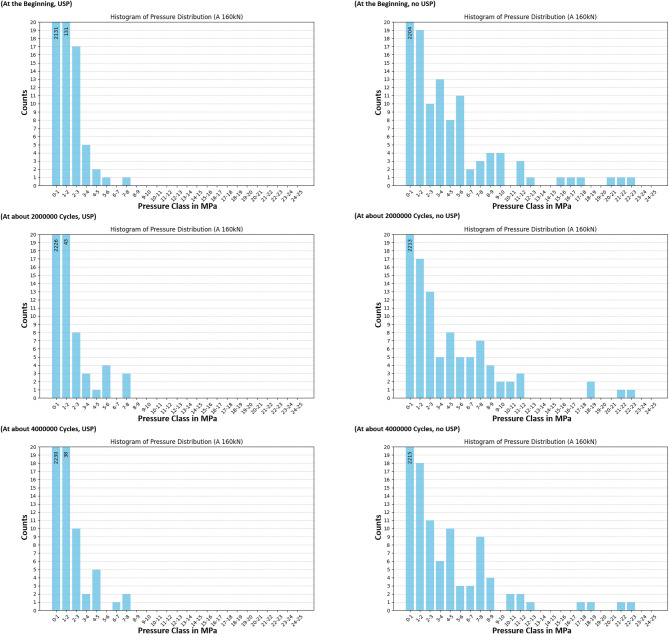
Histogram of pressure distribution for sensor A at high load.

**Fig. 19:**
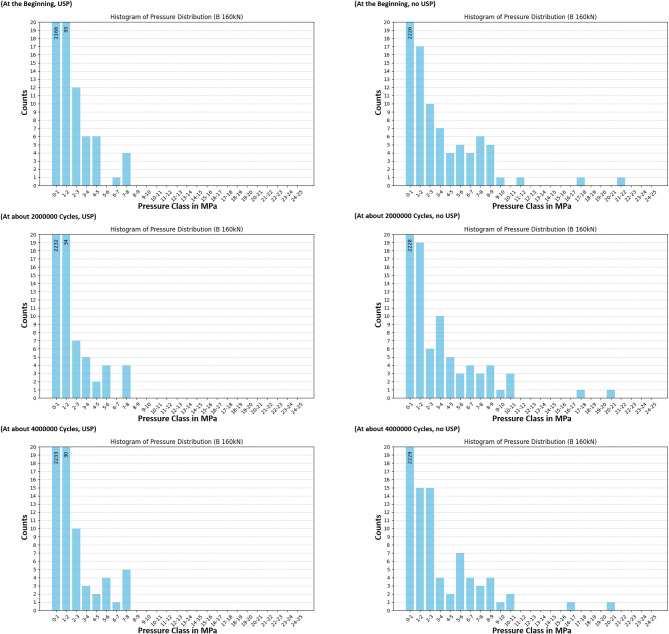
Histogram of pressure distribution for sensor B at high load.

**Fig. 20:**
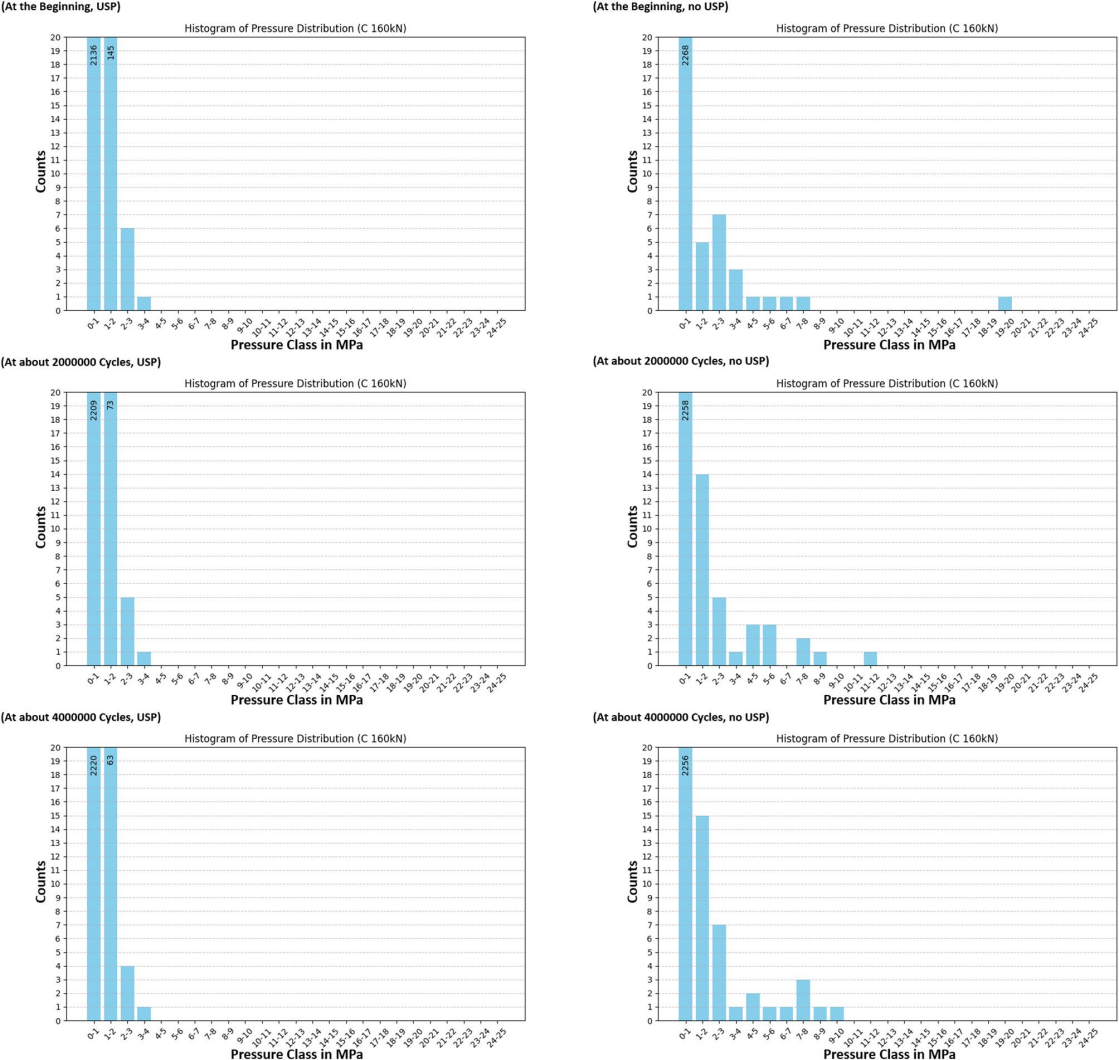
Histogram of pressure distribution for sensor C at high load.

The redistribution process is more clearly observed through the use of pressure class histograms, providing a more precise method for analyzing changes in pressure distribution under load. Figures 18 and 19, and Fig. 20 present the pressure distributions for sensors A, B, and C, respectively. The vertical axis represents the number of values within each pressure class, while the horizontal axis indicates the pressure classes, defined by their lower and upper bounds in MPa. The range of pressure classes was selected to cover the full spectrum of measured values, with class intervals chosen to be small enough to provide a detailed resolution for observing rearrangement effects. Due to the force application on the left side of the sleeper, only the sensors located on the left half were deemed significant for the analysis. The data shows a trend where higher pressure values decrease over time, resulting in a more uniform pressure distribution. This trend is evident in both the sleeper with under sleeper pads (USP) and the sleeper without USP, though it is more pronounced in the sleeper with USP. However, it is important to note that there are still instances where pressure may increase, as previously discussed.

### Dynamic of the sleeper

Changes in oscillation amplitude and movement patterns provide valuable insights into the dynamic behavior of the sleeper and its interaction with operational forces. For this analysis, 10 cycles were plotted from both the beginning phase and the end phase of the test. The movement profile may offer critical information about the sleeper’s dynamic response over time.

**Fig. 21:**
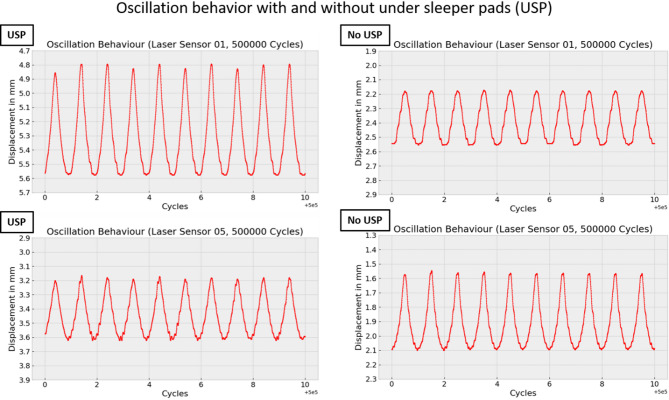
Oscillation behavior at the start under cyclic forces.

Figure 21 shows 10 cycles for laser sensor L1 and L5 after the first 500,000 cycles for the sleeper with and without USP. The observed waveform of the displacement deviates from the ideal sinusoidal shape of the applied external force. The turning point area in the unloaded state appears sharper compared with the loaded state, indicating asymmetric behavior. A second observed phenomenon is that the turning point in the unloaded state remains relatively constant over multiple cycles for the sleeper without under sleeper pads (USP). In contrast, the turning point for the sleeper with USP exhibits significant fluctuations. This difference may be due to the elastic properties of the USP material, which allows for more variability in the sleeper’s position when it is unloaded, while another explanation could be control errors of the force generator.

**Fig. 22:**
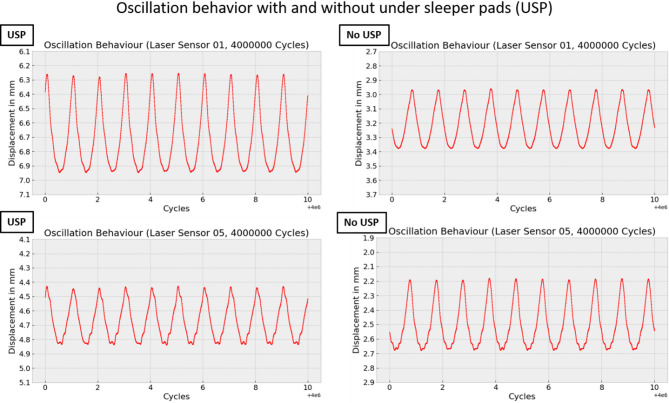
Oscillation behavior at the end under cyclic forces.

After four million cycles, the waveforms become smoother, and the fluctuation of the turning points disappears, as shown in Fig. 22.

## Discussion and conclusion

This study investigated the behavior of a turnout sleeper within a large-scale ballast box. Displacement sensors were utilized to monitor sleeper movement, while Getzner Sensor Sleeper technology was employed to map the pressure distribution beneath the sleeper. The results revealed significant differences in the behavior of sleepers with and without under sleeper pads (USP). Long-term settlement and pressure distribution varied notably between the two cases. Although the absolute movement of the sleeper was higher with USP due to the added elasticity, the use of USP resulted in significantly more uniform pressure distributions and substantially reduced pressure peaks.

The following list shows the key findings of this study:

- The sleeper’s settlement is primarily influenced by the load application point and the resulting pressure distribution, leading to greater settlement on the loaded side and significantly less on the passive side. Due to the asymmetric loading condition, the right side of the sleeper become insufficiently supported, causing it to lose contact with the ballast. The settlement behavior follows a degressive pattern, characterized by substantial initial settlement followed by progressively decreasing settlement rates in the later phases. Additionally, the use of under sleeper pads (USP) resulted in more even settlement behavior, reducing irregularities and discontinuities in the settlement process.
- The sleeper equipped with under sleeper pads (USP) experienced less bending as a result of the improved bedding condition. No significant voids or large unsupported areas were observed beneath this sleeper, which contributed to its more uniform support. In contrast, the sleeper without USP rested on discrete bearing points caused by large unsupported zones underneath. These act as abutments, inducing bending when loads are applied.
- The presence of under-sleeper pads (USP) resulted in a more uniform pressure distribution with notably reduced pressure peaks and gentler pressure gradients. Over time, the pressure distribution becomes more uniform for both sleepers with and without under sleeper pads (USP). However, this effect is more pronounced in sleepers equipped with USP, indicating a greater improvement in load distribution. The contact between sleeper and ballast remained stable over time, with no voids or unsupported areas developing.
- Sleepers with under sleeper pads (USP) show larger oscillation amplitudes due to the material’s elasticity. While the amplitude remains stable for sleepers without USP, it changes significantly at the beginning for those with USP, likely due to the plastic compression of the pad, which is made of elasto-plastic polyurethane. Over time, the sleeper’s movement becomes smoother, and fluctuations at the turning points during the unloaded state diminish, indicating a more stable and consistent response.
- The maximum pressure measured beneath sleepers equipped with USP was approximately 8 MPa. In contrast, sleepers without USP reached a maximum pressure of about 23 MPa—nearly three times higher. Only a few discrete contact points reached such high pressure levels. Most contact areas experienced significantly lower pressures, and the majority of points showed no contact at all.

During the experiment, it was noticed that the USP material displays time-dependent behavior, expanding again slowly after compression under loading. Therefore, the total sleeper settlement of the sleeper is a combination of USP deformation and ballast settlement, where only the latter is permanent. The measured vertical displacement which includes both ballast settlement and USP deformation, cannot be separated within the current test setup. Due to the continuous cyclic loading, the USP material did not have sufficient time to recover its original shape between load cycles, resulting in a combined, non-differentiated deformation response. In order to distinguish between a temporary change in track height due to USP compression and permanent settlement due to ballast settlement, further testing and modeling need to be performed.

Further, the oscillation behavior of the sleeper with USP showed that the waveform of the displacement becomes smoother and fluctuations of the turning points disappeared. This might be due to the elastic pad material being compressed to the point where it has hardened significantly, losing its original range of motion. As a result, the reduced range of motion leads to a decrease in oscillation amplitude and increased stiffness. A second possible explanation is that, after a large number of load cycles, the sleeper becomes more securely embedded in the ballast, with better contact. This occurs because the micro-movements induced by repeated loading cause the individual ballast stones to realign, resulting in a more compact and stable configuration. This improved alignment of the ballast particles leads to better load distribution and smoother deformation under subsequent cycles. The observed fluctuations may also be attributed to control inaccuracies in the force generation system.

Hydraulic actuators used in laboratory settings are unable to replicate the dynamic impact loading conditions typically observed in real-world scenarios. As a result, ballast box tests that employ hydraulic force generators can provide only a qualitative understanding of the mechanical interaction between ballast and sleeper, rather than fully capturing the dynamic response observed under real-world conditions.

In real-world track conditions, settlement along the track exhibits variability due to variations in the ballast layer. This results in the development of “hanging sleepers,” where some sleepers are suspended above the ballast due to uneven settlement along the track, particularly in turnout areas. These uneven settlements create gaps under certain sleepers, leading to irregular support conditions.

The experiment primarily yielded qualitative insights into the behavior of turnout sleepers. The quantitative results, however, are highly dependent on various parameters. As is known, the track bed conditions vary at each point, leading to differences in exact movement, settlement behavior, and pressure distributions. To obtain more detailed and transferable results, further investigations under real-world conditions are necessary. In addition to displacement measurements and long-term monitoring of sleeper and ballast conditions, the use of sensor-integrated rail pads to monitor load transfer from the rail to the sleeper over extended periods would be a particularly valuable aspect for future studies. Field measurements in particular can offer more detailed and realistic results, potentially informing improvements in laboratory tests or parameter studies. Key factors such as ballast properties, sleeper design, and the characteristics of under sleeper pads (USPs) significantly influence settlement behavior and ballast degradation. Therefore, future research should focus on detailed investigations of these parameters. The resulting data can support the development and validation of numerical models, enabling more accurate and efficient prediction of settlement behavior in track systems.

From an economic perspective, the use of under sleeper pads (USP) can offer significant long-term benefits for railway infrastructure by extending maintenance intervals, which are often complex and cost-intensive. By improving the contact conditions between sleeper and ballast, USPs promote a more uniform load distribution and reduce high local stresses. Additionally, impact forces can be mitigated through the cushioning effect and lower stiffness of the USP material. These effects can contribute to reduced maintenance requirements, longer service life of track components, and a lower risk of damage. Although the initial investment in USPs is higher compared to conventional track structures, the potential savings in maintenance costs and the reduction of track closures for repair work can outweigh the upfront expenses, particularly in high-traffic or maintenance-critical areas such as turnouts and transition zones. To support a more accurate and realistic cost-benefit analysis, long-term field studies are needed, incorporating a range of USP materials and geometries. Such studies would allow for a comprehensive assessment of endurance improvements under real-world conditions.

Nevertheless, the study provided novel and deep insights into the load transfer mechanisms in a turnout and provides a good baseline for future research, track component development and maintenance strategies.

## Data Availability

The datasets generated and analysed during the current study are not publicly available due to project guidelines and proprietary restrictions but are available from the corresponding author on reasonable request.

## References

[1] Kumar, N. et al. Micro-mechanical investigation of railway ballast behavior under Cyclic loading in a box test using DEM: effects of elastic layers and ballast types. *Granul. Matter* **21** (4), 106. 10.1007/s10035-019-0956-9 (2019). 31708679 10.1007/s10035-019-0956-9 PMC6813763

[2] Lackenby, J., Indraratna, B. & McDowell, G. Effect of confining pressure on ballast degradation and deformation under Cyclic triaxial loading. *Géotechnique* **57** (6), 527–536. 10.1680/geot.2007.57.6.527 (2007).

[3] Abadi, T., Le Pen, L., Zervos, A. & Powrie, W. Improving the performance of railway tracks through ballast interventions. *Proc. Inst. Mech. Eng. Part F: J. Rail Rapid Transit.* **232** (2), 337–355. 10.1177/0954409716671545 (2018).

[4] Sd, K., Karimullah Hussaini, B., Indraratna, J. & Vinod, S. An experimental investigation on the deformation and degradation behaviour of geogrid-reinforced ballast (2023).

[5] Hamarat, M., Papaelias, M., Silvast, M. & Kaewunruen, S. The effect of unsupported sleepers/bearers on dynamic phenomena of a railway turnout system under impact loads. *Appl. Sci.* **10** (7), 2320. 10.3390/app10072320 (2020).

[6] Lundqvist, A. & Dahlberg, T. Load impact on railway track due to unsupported sleepers. *Proc. Institution Mech. Eng. Part. F: J. Rail Rapid Transit.* **219** (2), 67–77. 10.1243/095440905X8790 (2005).

[7] Zhang, X., Zhao, C., Zhai, W., Shi, C. & Feng, Y. Investigation of track settlement and ballast degradation in the high-speed railway using a full-scale laboratory test. *Proc. Institution Mech. Eng. Part. F: J. Rail Rapid Transit.* **233** (8), 869–881. 10.1177/0954409718812231 (2019).

[8] Dai, J., Keng Ang, K. & Jiang, D. Moving element analysis of high-speed rail system accounting for hanging sleepers. *MATEC Web Conf.* **148**, 5007. 10.1051/matecconf/201814805007 (2018).

[9] Indraratna, B., Thakur, P. K. & Vinod, J. S. Experimental and numerical study of railway ballast behavior under Cyclic loading. *Int. J. Geomech.* **10** (4), 136–144. 10.1061/(ASCE)GM.1943-5622.0000055 (2010).

[10] Estaire, J., Cuellar, V., Pardo de Santayana, F. & Santana, M. Testing railway tracks at 1 1 scale at CEDEX Track Box (2023).

[11] Sun, Q. D., Indraratna, B. & Nimbalkar, S. Deformation and degradation mechanisms of railway ballast under high frequency Cyclic loading. *J. Geotech. Geoenviron Eng.* **142**, 1. 10.1061/(ASCE)GT.1943-5606.0001375 (2016).

[12] Sun, Q. D., Indraratna, B. & Nimbalkar, S. Effect of Cyclic loading frequency on the permanent deformation and degradation of railway ballast. *Géotechnique* **64** (9), 746–751. 10.1680/geot.14.T.015 (2014).

[13] Wang, X., Pu, J., Wu, P. & Chen, M. Modeling of coupling mechanism between ballast bed and track structure of High-Speed railway. *Math. Probl. Eng.* **2020** (1), 1–12. 10.1155/2020/9768904 (2020).

[14] Holtzendorff, K. Untersuchung des Setzungsverhaltens von Bahnschotter und der Hohllagenentwicklung auf Schotterfahrbahnen (2023).

[15] Abadi, T., Le Pen, L., Zervos, A. & Powrie, W. A review and evaluation of ballast settlement models using results from the Southampton railway testing facility (SRTF). *Procedia Eng.* **143**, 999–1006. 10.1016/j.proeng.2016.06.089 (2016).

[16] Saussine, G., Quezada, J. C., Breul, P. & Radjai, F. *Railway Ballast Settlement: A New Predictive Model. Jonesboro Ga. (P.O. Box 1507 Jonesboro 30236* (Ancestors Unlimited, 1986).

[17] Shenton, M. J., Deformation of railway ballast under repeated loading conditions. In *Railroad Track Mechanics and Technology* 405–425 (Elsevier, 1978).

[18] Quezada, J. C., Saussine, G., Breul, P. & Radjaï, F. Predicting the settlement of coarse granular materials under vertical loading. *Sci. Rep.* **4**, 5707. 10.1038/srep05707 (2014). 25028053 10.1038/srep05707 PMC4099977

[19] Pircher, P. *A DEM Model for Elastic Sleepers to Study Dynamic Railway Track Behaviour* (Montanuniversität Leoben, 2021).

[20] Erol, T. et al. *Discrete Element Modeling of Railroad Ballast Behavior* (Springer, 2024).

[21] Buddhima Indraratna, F. A. S. C. E. et al. Experimental and numerical study of railway ballast behavior under cyclic loading. *School Civ. Min. Environ. Eng.* (2023).

[22] Anderson, W. & Fair, P. Behavior of railroad ballast under monotonic and cyclic loading. *J. Geotech. Geoenviron. Eng.* (2008).

[23] Suhr, B., Marschnig, S. & Six, K. Comparison of two different types of railway ballast in compression and direct shear tests: experimental results and DEM model validation. *Granul. Matter*. **20** (4), 70. 10.1007/s10035-018-0843-9 (2018). 30532652 10.1007/s10035-018-0843-9 PMC6245121

[24] Nurmikolu, A., Kerokoski, O., Rantala, T. & Viitala, T. Cyclic loading tests of concrete sleepers with varying ballast condition. *Joint Rail Conf.* **2010**, 257–265. 10.1115/JRC2010-36147 (2010).

[25] Shenton, M. J. Ballast deformation and track deterioration (2023).

[26] Kennedy, J. A full-scale laboratory investigation into railway track substructure performance and ballast reinforcement (2024).

[27] Lackenby, J., Indraratna, B., McDowell, G. & Christie, D. Effect of confining pressure on the degradation of ballast under cyclic loading. *Geotechnique* (2005).

[28] Ishikawa, T., Sekine, E. & Miura, S. Cyclic deformation of granular material subjected to moving-wheel loads. *Can. Geotech. J.* **48** (5), 691–703. 10.1139/t10-099 (2011).

[29] Harry, E. & Stewart, M. School of Civ. and Environ. Engrg., Cornell Univ., Ithaca, NY 14853‐3501. In *Permanent Strains from Cyclic Variable-Amplitude Loadings* (2024).

[30] Abadi, T., Le Pen, L., Zervos, A. & Powrie, W. Effect of sleeper interventions on railway track performance. *J. Geotech. Geoenviron Eng.* **145**, 4 . 10.1061/(ASCE)GT.1943-5606.0002022 (2019).

[31] Abadi, T., Le Pen, L., Zervos, A. & Powrie, W. Improving the performance of railway tracks through ballast interventions. *Proc. Institution Mech. Eng. Part. F: J. Rail Rapid Transit.* **232** (2), 337–355. 10.1177/0954409716671545 (2018).

[32] Mechanics of railway ballast behaviour (2004).

[33] Stabilisation of rail tracks and underlying soft soil formations (2024).

[34] Indraratna et al. Geotechnical properties of ballast and the role of geosynthetics in rail track stabilisation-annotated (2006).

[35] A laboratory study of railway ballast behaviour under traffic loading and tamping maintenance (2024).

[36] Manalo, A., Aravinthan, T., Karunasena, W. & Stevens, N. Analysis of a typical railway turnout sleeper system using grillage beam analogy. *Finite Elem. Anal. Des.* **48** (1), 1376–1391. 10.1016/j.finel.2011.08.007 (2012).

[37] Zakeri, J. A., Fattahi, M., Nouri, M. & Janatabadi, F. Influence of rail pad stiffness and axle loads on dynamic responses of Train-track interaction with unsupported sleepers. *Period Polytech. Civil Eng.* **64** (2), 524–534. 10.3311/PPci.14826 (2020).

[38] Zhu, J. J., Ahmed, A. K., Rakheja, S. & Khajepour, A. Development of a vehicle–track model assembly and numerical method for simulation of wheel–rail dynamic interaction due to unsupported sleepers. *Veh. Syst. Dyn.* **48** (12), 1535–1552. 10.1080/00423110903540751 (2010).

[39] Kaewunruen, S., Ishida, T. & Remennikov, A. M. Dynamic performance of concrete turnout bearers and sleepers in railway switches and crossings. *Adv. Civ. Eng. Matls* **7** (3), 20170103. 10.1520/ACEM20170103 (2018).

[40] Namura, A., Kohata, Y. & Miura, S. Effect of sleeper size on ballasted track settlement. *Q. Rep. Rtri* (2004).

[41] Chi, Y., Xiao, H., Zhang, Z., Fang, S. & Wang, H. Discrete element analysis on mechanical properties of ballast bed by tamping in railway turnout areas. *J. Comput. Nonlinear Dyn.* **17**, 11. 10.1115/1.4055429 (2022).

[42] Kaewunruen, S. & Remennikov, A. M. Investigation of free vibrations of voided concrete sleepers in railway track system. *Proc. Institution Mech. Eng. Part. F: J. Rail Rapid Transit.* **221** (4), 495–507. 10.1243/09544097JRRT141 (2007).

[43] Bednarek, W. A. Full-Scale field experimental investigation on the intended irregularity of CWR track in vertical plane. *Energies* **14** (22), 7477. 10.3390/en14227477 (2021).

[44] Shi, J., Chan, A. H. & Burrow, M. P. N. Influence of unsupported sleepers on the dynamic response of a heavy haul railway embankment. *Proc. Institution Mech. Eng. Part. F: J. Rail Rapid Transit.* **227** (6), 657–667. 10.1177/0954409713495016 (2013).

[45] Sadeghi, J. Field investigation on dynamics of railway track pre-stressed concrete sleepers. *Adv. Struct. Eng.* **13** (1), 139–151. 10.1260/1369-4332.13.1.139. (2010).

[46] Abadi, T., Le Pen, L., Zervos, A. & Powrie, W. Measuring the area and number of ballast particle contacts at Sleeper-Ballast and ballast-Subgrade interfaces. *Int. J. Railw Tech.* **4** (2), 45–72. 10.4203/ijrt.4.2.3 (2015).

[47] Steiner, K. *Prager—2012—Druckausbreitung von belasteten Eisenbahnschwellen im Gleisschotter-annotated* (2023).

[48] Alabbasi, Y. & Hussein, M. Large-scale triaxial and box testing on railroad ballast: a review. *SN Appl. Sci.* **1** (12), 1–23. 10.1007/s42452-019-1459-3 (2019).

[49] Al Shaer, A., Duhamel, D., Sab, K., Foret, G. & Schmitt, L. Experimental settlement and dynamic behavior of a portion of ballasted railway track under high speed trains. *J. Sound Vib.* **316** (1–5), 211–233. 10.1016/j.jsv.2008.02.055 (2008).

[50] Marschnig, S., Ehrhart, U. & Offenbacher, S. Long-Term behaviour of padded concrete sleepers on reduced ballast bed thickness. *Infrastructures* **7** (10), 132. 10.3390/infrastructures7100132 (2022).

[51] Le Pen, L., Watson, G., Hudson, A. & Powrie, W. Behaviour of under sleeper pads at switches and crossings—field measurements. *Proc. Inst. Mech. Eng. F J. Rail Rapid Transit.* **232** (4), 1049–1063. 10.1177/0954409717707400 (2018). 30662165 10.1177/0954409717707400 PMC6319517

[52] Kraśkiewicz, C., Oleksiewicz, W., Płudowska-Zagrajek, M. & Piotrowski, A. Testing procedures of the Under Sleeper Pads applied in the ballasted rail track systems. *MATEC Web Conf.* **196**, 2046. 10.1051/matecconf/201819602046 (2018).

[53] Smirnov, V. Dynamic analysis of railway track with vibration isolated booted sleepers. *MATEC Web Conf.* **196**, 1045. 10.1051/matecconf/201819601045 (2018).

[54] Navaratnarajah, S. K. & Indraratna, B. Use of rubber Mats to improve the deformation and degradation behavior of rail ballast under Cyclic loading. *J. Geotech. Geoenviron Eng.* **143**, 6. 10.1061/(ASCE)GT.1943-5606.0001669 (2017).

[55] Wickramarachchi, W., Chamindi, J. & Jayasuriya, I. *Nimbalkar – 2015 - Analysis of the Performance of Under Sleeper Pads-A Critical Review-annotated* (2015).

[56] Gräbe, P., Mtshotana, B., Sebati, M. & Thünemann, E. The effects of under-sleeper pads on sleeper-ballast interaction. *J. S Afr. Inst. Civ. Eng.* **58** (2), 35–41. 10.17159/2309-8775/2016/v58n2a4 (2016).

[57] Armin, B. Wirkungsweise des Schotters im Gleis unter verschiedenen Randbedingungen (2023).

[58] Kumar, N., Kossmann, C., Scheriau, S. & Six, K. An efficient physical-based method for predicting the long-term evolution of vertical railway track geometries. *Proc. Institution Mech. Eng. Part. F: J. Rail Rapid Transit.* **236** (4), 447–465. 10.1177/09544097211024803 (2022).

[59] Schneider, P., Bolmsvik, R. & Nielsen, J. C. O. In situ performance of a ballasted railway track with under sleeper pads. *Proc. Inst. Mech. Eng. Part F: J. Rail Rapid Transit.* **225** (3), 299–309. 10.1177/2041301710392479 (2011).

[60] Navaratnarajah, S. K., Indraratna, B. & Nimbalkar, S. Application of shock Mats in rail track foundation subjected to dynamic loads. *Procedia Eng.* **143**, 1108–1119. 10.1016/j.proeng.2016.06.152 (2016).

[61] Quirchmair, M., Loy, H., Sehner, M. & Pümpel, M. ZEVrail_Heft 11_12 2023 Schotterschonung mit Schwellensohlen. *Zevrail* **2023**, 147 (2023).

[62] Sehner, M., Augustin, A., Quirchmair, M. & Loy, H. 2023_ZEV_Sensor sleeper technology_view between the sleeper and the ballast. *Zevrail* **2023**, 147 (2023).

