# Correction: Behavior of turnout sleepers in a large-scale ballast box test

**DOI:** 10.1038/s41598-025-32461-5

**Published:** 2025-12-23

**Authors:** Gernot Grohs, Paul Pircher, Martin Quirchmair, Harald Loy, Klaus Six, Ferdinand Pospischil

**Affiliations:** 1 Institute of Railway Infrastructure Design, Graz University of Technology, Graz, Austria; 2 Virtual Vehicle Research GmbH, Graz, Austria; 3 Getzner Werkstoffe GmbH, Bürs, Austria; 4 Department of Infrastructure, Unit of Intelligent Transport Systems, University of Innsbruck, Innsbruck, Austria

Correction to: *Scientific Reports* 10.1038/s41598-025-01751-3, published online 14 May 2025

The original version of this Article contained an error in Fig. 14, where in the data analysis, the sign was used incorrectly, causing the uplift to appear smaller at higher forces. The incorrect Figure 14 along with its caption is provided below.

**Figure 14:**
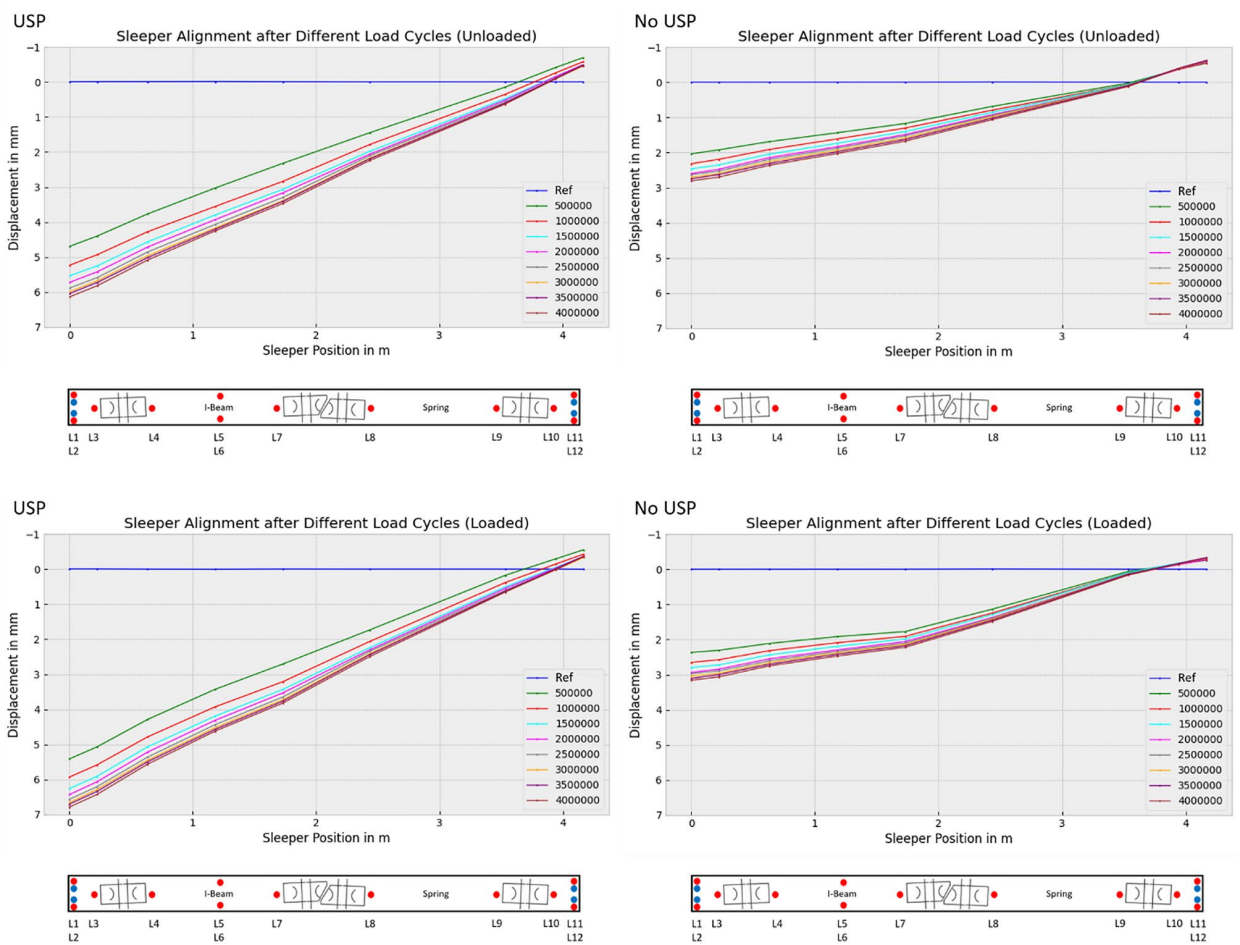
Alignment of turnout sleeper under varying load conditions after different load cycles.

The original Article has been corrected.

